# Kinematics-Based Assessment of Reaching and Grasping Movements in LRN Ablated Animals Identifies a Role for the LRN in Endpoint Stabilization and Reach Timing

**DOI:** 10.1523/ENEURO.0147-26.2026

**Published:** 2026-09-11

**Authors:** Gavin Thomas Koma, Joshua D. Ross, Thomas J. Campion, Jacquelynn Rajavong, Yuanchao Zhang, George M. Smith, Andrew J. Spence

**Affiliations:** ^1^Temple University, Philadelphia, Pennsylvania 19122; ^2^Lewis Katz School of Medicine, Philadelphia, Pennsylvania 19140; ^3^Children’s Hospital of Philadelphia, Philadelphia, Pennsylvania 19104

**Keywords:** kinematics, lateral reticular nucleus, reaching

## Abstract

The lateral reticular nucleus (LRN) is positioned to relay motor-related information to cerebellar circuits, but its direct contribution to skilled reaching and grasping remains unclear. Here, we examined skilled forelimb behavior in intact adult female Long–Evans rats after bilateral, cell type-targeted LRN ablation using single-pellet reaching, qualitative scoring, and quantitative three-dimensional kinematic analysis. We assessed whether LRN loss disrupted gross limb transport, endpoint precision, trial-to-trial consistency, and reach timing across pre- and post-surgical recording sessions. LRN-ablated animals continued to generate broadly recognizable pellet-directed reaches, indicating that the LRN is not required for basic reach production. However, group differences emerged in restricted portions of the reach trajectory and were most prominent in pellet-directed endpoint control. Experimental animals showed broader endpoint covariance, greater endpoint spread, and increased trial-to-trial variability, indicating less precise and less reproducible forelimb placement relative to the pellet. These effects were not explained by a single fixed spatial offset, but instead reflected reduced endpoint stabilization accompanied by selective coordinate-specific changes, including altered paw height. Reach duration was also altered, but these timing differences emerged later and were less prominent than the spatial endpoint deficits. Together, these findings suggest that the LRN contributes primarily to the refinement, stabilization, and timing of skilled forelimb movements rather than to gross reach initiation or limb transport. This work provides a direct in vivo model for studying LRN-dependent control of skilled reaching and highlights the value of kinematic analysis for detecting subtle movement deficits beyond retrieval success alone.

## Significance Statement

The lateral reticular nucleus (LRN) relays motor-related information to cerebellar circuits, but its role in skilled reaching remains unclear. Using bilateral, cell type-targeted LRN ablation with single-pellet reaching and three-dimensional kinematic analysis in adult rats, we found that LRN loss did not abolish pellet-directed reaching. Instead, ablation reduced endpoint precision, increased trial-to-trial variability, and produced later changes in reach duration. These findings suggest that the LRN contributes primarily to the refinement and stabilization of skilled forelimb movements rather than basic reach initiation, and show how kinematic analysis can reveal movement deficits not captured by retrieval success alone.

## Introduction

Dexterous forelimb movements such as reaching, grasping, and food retrieval require coordinated control of limb transport, distal positioning, fine digit placement, and movement refinement (Whishaw et al., 1992). Rodent single-pellet reaching provides a well-established model for studying these processes because reach-to-grasp movements share key organizational features with primate and human forelimb behaviors while improving gradually with practice (Whishaw et al., 1992; Klein et al., 2012; Bova et al., 2021). Traditional assessments have relied heavily on pellet retrieval success or qualitative scoring of movement components, which are useful for identifying overt performance changes but provide limited information about the kinematic organization, refinement, or disruption of the movement itself (Whishaw et al., 2008; Klein et al., 2012; Parmiani et al., 2019; Bova et al., 2021). Recent advances in pose estimation and three-dimensional kinematic analysis now allow more detailed measurement of trajectory shape, endpoint variability, and reach timing (Mathis et al., 2018; Nath et al., 2019; Parmiani et al., 2019). Markerless pose estimation approaches have also been applied successfully to reach and grasp behaviors across species, including rodent forelimb tasks, where multi-camera tracking and three-dimensional reconstruction can resolve detailed limb kinematics without requiring physical markers (Bova et al., 2021; Karashchuk et al., 2021). These approaches are particularly useful for skilled reaching because they allow trial-level quantification of limb trajectory, endpoint position, and movement variability during naturalistic reach-to-grasp behavior. These approaches have revealed movement features that may change independently across training and may depend on distinct neural circuits during acquisition and mature task performance (Nica et al., 2018; Bova et al., 2021).

One circuit positioned to support skilled forelimb refinement is the lateral reticular nucleus (LRN) and its associated propriospinal and cerebellar connections (Alstermark and Ekerot, 2013, 2015; Jiang et al., 2015). The LRN is a precerebellar nucleus that relays motor-related signals to the cerebellum, where they may be integrated with sensory feedback to support online movement correction (Alstermark and Ekerot, 2013, 2015; Azim and Alstermark, 2015). Within the cervical spinal cord, C3–C4 propriospinal neurons project both to forelimb-related motor circuitry and to the LRN, forming an anatomical substrate through which descending motor commands may be conveyed as an internal copy of the motor plan (Azim et al., 2014b; Azim and Alstermark, 2015; Jiang et al., 2015). These neurons receive convergent descending input and have been implicated in the control and calibration of skilled forelimb movements (Azim et al., 2014b; Azim and Alstermark, 2015).

Functional studies further support the idea that LRN-associated pathways contribute to forelimb motor control (Kinoshita et al., 2012; Azim et al., 2014b; Alstermark and Ekerot, 2015). Manipulation of C3–C4 propriospinal neurons produces errors in forelimb kinematics, and disruption of related circuitry has been linked to deficits in reaching and grasping behaviors (Kinoshita et al., 2012; Azim et al., 2014b; Azim and Alstermark, 2015). Together, these observations suggest that the C3–C4 propriospinal-LRN-cerebellar pathway may contribute to an efference-copy mechanism in which motor-related signals are compared with ongoing sensory information to support real-time error correction (Azim et al., 2014b; Alstermark and Ekerot, 2015; Azim and Alstermark, 2015; Huma and Maxwell, 2015; Jiang et al., 2015). The LRN may also help signal progression through the movement, providing information that could regulate the timing or gating of successive reach and grasp components (Azim et al., 2014a; Alstermark and Ekerot, 2015; Azim and Alstermark, 2015).

Despite this anatomical and physiological evidence, the specific behavioral consequences of direct LRN ablation during skilled reaching remain insufficiently defined in vivo. In particular, it is unclear whether loss of LRN function primarily disrupts gross forelimb trajectory control, fine endpoint precision, reach timing, or the coordination between these components. Addressing this question requires quantitative behavioral analysis capable of resolving detailed kinematic changes beyond pellet retrieval success alone (Klein et al., 2012; Mathis et al., 2018; Nath et al., 2019; Parmiani et al., 2019; Bova et al., 2021).

In the present study, we examined skilled forelimb control in intact adult female Long–Evans rats using bilateral, cell type-targeted LRN ablation in vGlut1-Cre transgenic animals. We paired single-pellet reaching with pellet retrieval success, qualitative reach scoring, and quantitative three-dimensional kinematic analyses of forelimb movement patterning. Specifically, we assessed features not captured by endpoint success alone, including endpoint covariance structure, forelimb trajectory shape, reach duration, endpoint position (Fig. 5*B*–*D*), and trial-to-trial variability across recording days. Given the proposed role of the LRN in relaying motor-related signals to cerebellar circuits involved in movement calibration and online correction, we hypothesized that bilateral LRN ablation would produce measurable abnormalities in the pattern, consistency, and precision of skilled reaching. The objective of this study was therefore to determine how the LRN contributes to normal skilled forelimb kinematics and to define the specific aspects of reaching behavior that depend on its integrity.

## Materials and Methods

### Subjects

**Figure 1.**
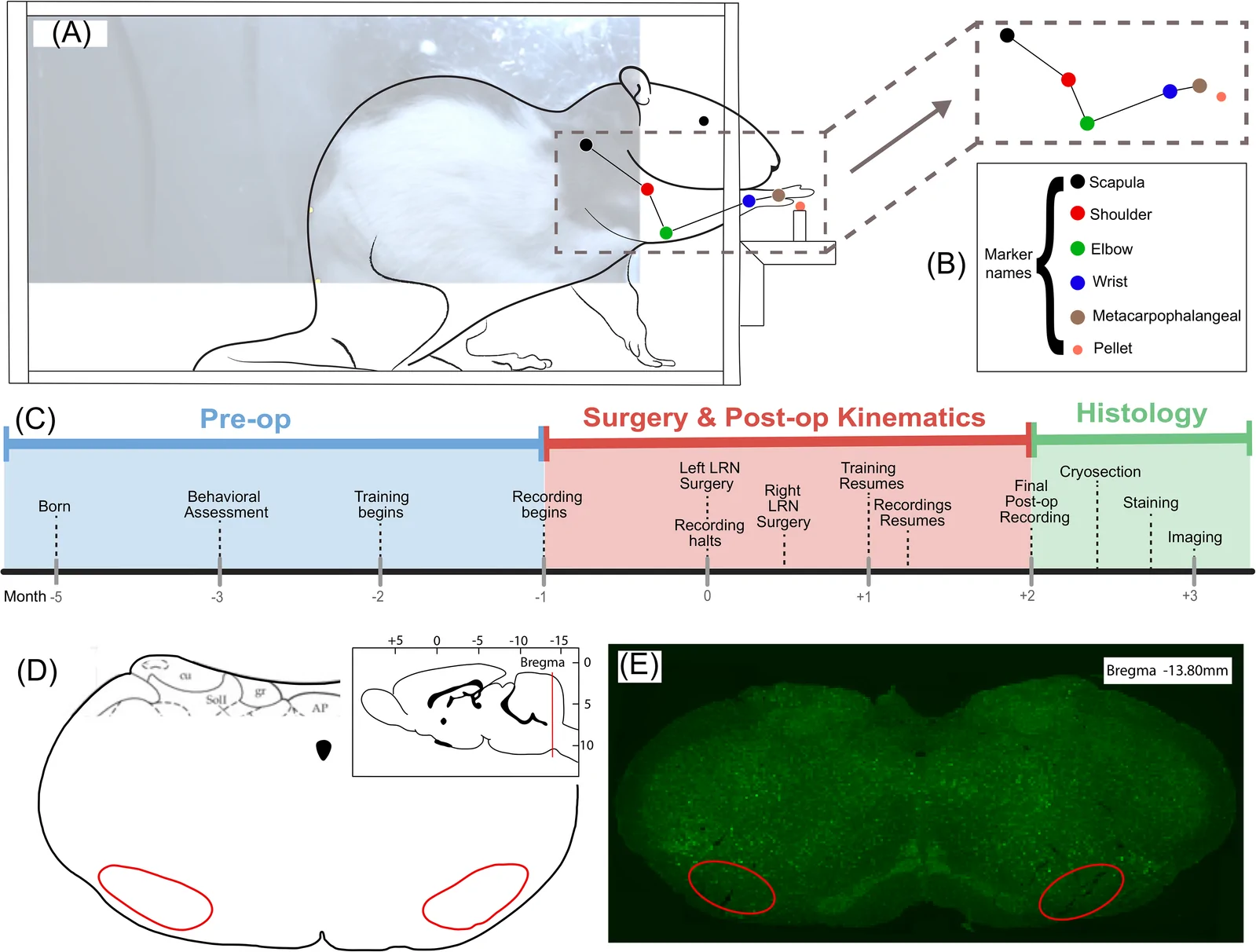
Apparatus, tracked kinematic landmarks, experimental timeline, anatomical location of the LRN, and histological confirmation of targeting. ***A***, Representative lateral view of a rat performing the pellet reaching task, with the tracked forelimb and body landmarks overlaid. ***B***, Marker set used for kinematic reconstruction, including the scapula, shoulder, elbow, wrist, metacarpophalangeal joint, and pellet location. ***C***, Experimental timeline showing the pre-operative period, bilateral LRN surgeries, post-operative kinematic recording period, and subsequent histological processing. ***D***, Schematic coronal atlas section illustrating the bilateral LRN target region. Red outlines contain the parvocellular (LRtPC), magnocellular (LRtMC/LRt), and subtrigeminal (LRtS5) divisions of the LRN. The inset indicates the rostrocaudal level used for histological verification. Atlas adapted from *The Rat Brain in Stereotaxic Coordinates* (Paxinos and Watson, 2013). ***E***, Representative coronal histological section showing bilateral lesion sites within the LRN, indicated by red outlines. NeuroTrace 435/455 fluorescent Nissl signal is pseudocolored green for visualization. Reduced Nissl signal within the outlined LRN regions indicates loss of neuronal cell bodies following FLEX-DTA-mediated ablation. RNAscope validation of dense vGlut1 expression within the LRN is provided in Extended Data Figure 1-1, and validation of comparatively sparse GABA expression within the LRN is provided in Extended Data Figure 1-2.

**Figure 2.**
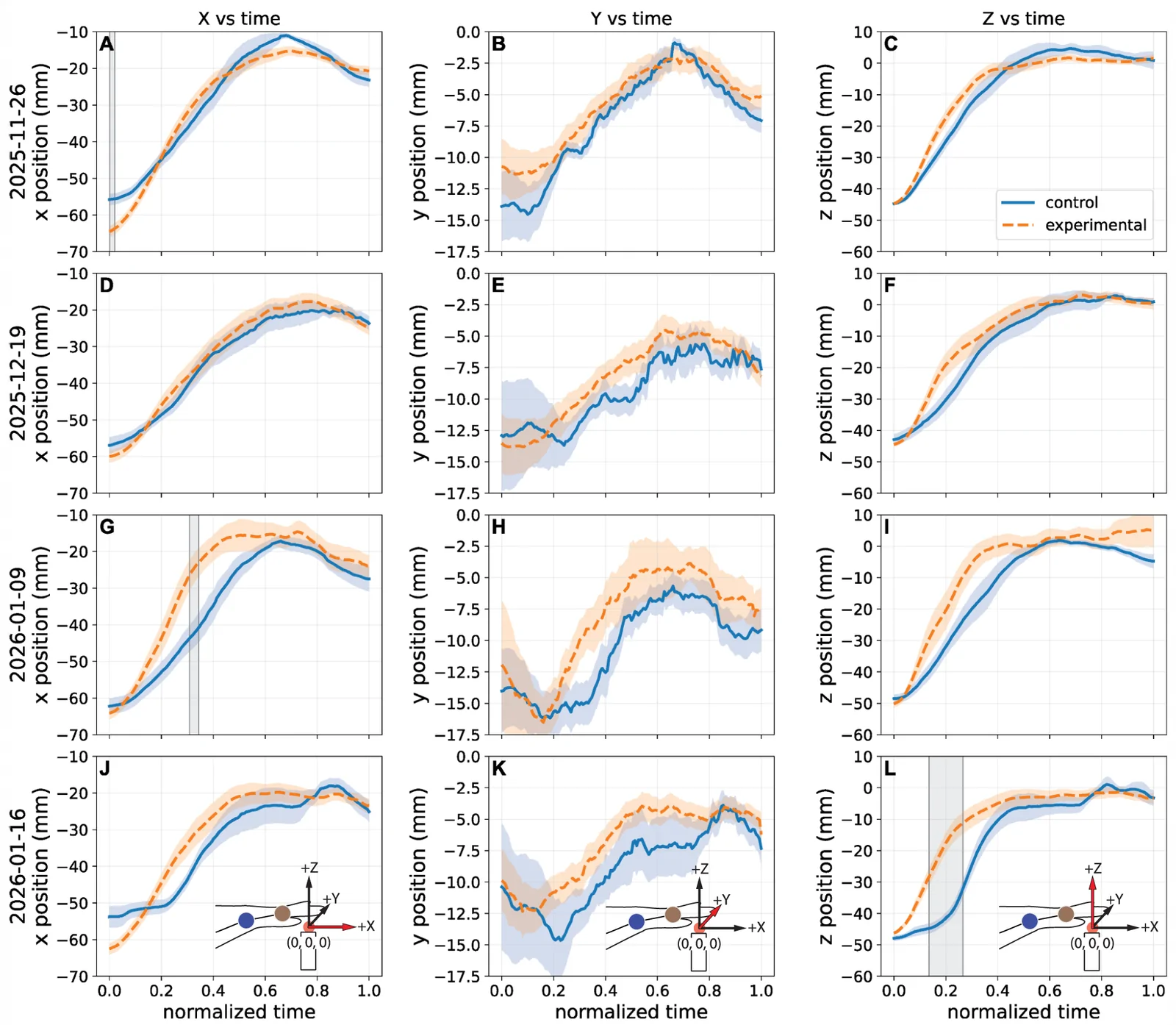
Comparison of Control versus Experimental wrist 3D trajectories. Panels ***A***–***C***, correspond to day −12, pre-surgery, ***D***–***F***, to day +11, ***G***–***I***, to day +32, and ***J***–***L***, to day +39, the latter three being post-ablation. Within each date, plots show wrist *x*, *y*, and *z* position, respectively, against normalized reach time. Please note orientation images in the bottom row illustrating the coordinate system definition relative to the reaching task; highlighted red arrows correspond to the axis of the coordinate being graphed in that column. Curves show the trial-level mean and shaded bands indicate standard error of the mean (SEM), with each reach analyzed as an individual trial. Across all dates, the overall shape of the wrist trajectory was largely preserved between groups, although axis-specific differences emerged during discrete portions of the reach. Reach-level statistical parametric mapping (SPM; *α* = 0.05) detected significant group differences in *x* on day −12 (***A***; normalized time 0.0000–0.0206, *p* = 0.0483^*a*^) and day +32 (***G***; 0.3076–0.3439, *p* = 0.0435^*a*^) and in *z* on day +39 (***L***; 0.1342–0.2662, *p* = 0.0028^*a*^), whereas the remaining comparisons were not significant. Gray vertical lines denote significant SPM clusters. Corresponding animal-level three-dimensional elbow-angle waveforms and SPM comparisons are provided in Extended Data Figure 2-1.

The rats used in this study were engineered to have a Cre knock-in at the vGlut1 (SLC17A7) locus. The vGlut1-Cre line was selected based on RNAscope validation showing dense vGlut1 expression within LRN neurons and comparatively little vGlut1 signal in the immediately surrounding medullary regions (Fig. 1-1). RNAscope analysis also showed comparatively sparse GABA expression within the LRN relative to surrounding tissue (Fig. 1-2). Together, these expression patterns supported the use of the vGlut1-Cre line and Cre-dependent AAV-mCherry-FLEX-DTA construct to direct DTA expression primarily to vGlut1-positive LRN neurons while limiting expression in surrounding medullary regions. Two litters of heterozygous vGlut1-Cre Long–Evans rats were bred using two female homozygous vGlut1-Cre rats and two male wild-type rats, producing an initial cohort of 18 animals. Fourteen animals, including nine females and five males, entered skilled reaching training. Six female animals that met the behavioral training criterion were selected for surgery. Only female animals underwent surgery and were included in the present experimental analysis to maintain consistency within the available vGlut1-Cre breeding cohort and to avoid introducing sex as an additional experimental variable in this initial study. Because sex was not included as an experimental variable, the present findings should be interpreted as specific to adult female Long–Evans rats. Their genotypes were confirmed via gel electrophoresis by gathering tail snips, digesting them, purifying the DNA and running PCR using the primer sets provided with the transgenic rats [Rat Slc17a7(Cre) Knock-In, LFKIR-211020-CHG-01, Cyagen]. Animals were housed in pairs under a 12:12 h light-dark cycle, with lights on at 6am EST [ZT0]. Behavioral testing was performed between ZT[6] and ZT[8]. All animal procedures were performed in accordance with the Temple University animal care committee’s regulations.

### Pre-operative behavioral assessment and reaching training

The baseline motor skill of these rats was measured using the Irvine, Beatties, and Bresnahan forelimb scale, grooming, and ladder walk assays at P54 (Metz and Whishaw, 2009; Al-Nasser, 2014; Irvine et al., 2014; Maze Engineers, Conduct Science, 2019). The rats then began reaching training at P71, which was comprised of three weekly sessions (MWF) of 15–20 attempts. Rats were habituated to the reaching chamber and food reward before formal training. During training sessions, animals were placed individually in the reaching apparatus and presented with single food pellets positioned outside the slit so that retrieval required a directed forelimb reach and grasp. Animals were allowed 15–20 pellet-directed attempts per session. Training occurred three times per week on Monday, Wednesday, and Friday. The preferred reaching limb was determined during early training and used for subsequent kinematic analyses. The recordings for kinematic analysis began at P108 and were done on the Friday of each week while training was still being performed Mondays and Wednesdays. These initial recordings can be considered the Pre-Operative Period. Rats were excluded from surgical enrollment if they were poor learners of the task, defined as performing below a 30% success rate and/or producing fewer than five attempts per session. Six female animals met these behavioral criteria and were selected for surgery.

### LRN injection surgery

To ablate the LRN, experimental adult Slc17a7-Cre rats were injected with AAV2-mCherry-FLEX-DTA (Addgene #58536; Uchida, 2014). Control Slc17a7-Cre rats were injected with the AAV2-hSyn-mCherry control construct (Addgene #114472; Deisseroth, 2018) under otherwise identical conditions. Of the fourteen animals that entered skilled reaching training, six female rats met the behavioral training criterion and were selected for surgery. These animals were randomly assigned to receive either the experimental FLEX-DTA construct or the control AAV2-hSyn-mCherry construct. Four animals received AAV2-mCherry-FLEX-DTA and two received AAV2-hSyn-mCherry. One FLEX-DTA-treated animal was subsequently excluded because of irregular reaching behavior, leaving five animals in the final kinematic cohort: three experimental animals and two control animals. Right and left injections were done a week apart due to high mortality of bilateral LRN injections on the same day. Rats were anesthetized with ketamine/xylazine anesthetic mixture. Once deeply sedated, the scalp was shaved, prepared for surgery, and secured in a stereotaxic frame. A 1.2 cm incision was made along the midline of the scalp and bregma identified. Bilateral holes (1 mm) were burred into the skull at the following coordinates: A/P: −13.7 mm, M/L: −/ + 1.6 mm, D/V: −10.5 mm. Only the medial/lateral were different to reflect the injection into either the right or left side. A 33-gauge needle attached to a 10 μl Hamilton syringe was lowered to the appropriate depth and virus injected into the LRN at a volume of 1.5 μl over 15 min. The needle remained in place for another 5 min and was slowly withdrawn. The incision was closed with surgical staples, and the contralateral LRN injection was performed one week later using the same procedure.

### Automatic 2D marker-tracking using DeepLabCut (DLC)

Reaching behavior was recorded using four synchronized high-speed Ximea cameras positioned laterally in pairs (two on each side) to provide complementary views of the reaching chamber for three-dimensional reconstruction. Videos were collected at 250 frames per second with a frame resolution of 2,048 × 700 pixels. Camera views were calibrated before three-dimensional reconstruction using DLT-based calibration procedures. DLC (version 2.1.8.2) was used to estimate the locations of the metacarpophalangeal (MCP) joint, wrist, elbow, shoulder, scapula from video recordings. Hindlimb joints were tracked for future analyses. Markers were successfully tracked using similar methodology as described by Mathis et al. (2018) and Nath et al. (2019). Forelimb joints were initially labeled manually and subsequently refined through the iterative correction of model predictions, resulting in a training dataset comprising of 10,550 frames. For network training, 95% of the dataset was allocated to the training set and 5% to the validation set using a ResNet-50-based architecture (He et al., 2015; Insafutdinov et al., 2016). A *p*-cutoff of 0.7 was applied to assess the reliability of joint marker detection. The finalized DLC model was then used to automatically track all remaining videos, and marker swaps or tracking jumps were manually corrected using a custom Python-based graphical review application. From these recordings, 163 reaches passed the initial behavioral segmentation and tracking-quality criteria. Of these, 157 reaches were represented across the four recording sessions included in the analyses reported here: 57 reaches at 12 d pre-surgery, 38 reaches at 11 d post-surgery, 26 reaches at 32 d post-surgery, and 36 reaches at 39 d post-surgery.

### Three-dimensional reconstruction and kinematic analyses

A custom Python code base was used to process DLC output into kinematic variables suitable for statistical analysis, informed by previously described marker-tracking methods (Maghsoudi et al., 2017). Two-dimensional joint positions from each camera view were combined with calibration matrices generated through DLTcal in MATLAB (R2025a) to reconstruct three-dimensional joint trajectories. Individual reaching trials were segmented using behaviorally relevant events identified from trial metadata, including paw lift off, passage through the slit, and trial end. Reaches were considered eligible for initial kinematic processing when the required behavioral start and end events were identified and reconstructed coordinate data were available across the analysis interval. The analyses reported here were restricted to eligible reaches from the four designated recording sessions. These events were used to define the temporal structure of the reach and to align movements across trials for comparison.

Derived kinematic variables were computed from the 3D trajectories and event-aligned reach segments. These included spatial features of the forelimb trajectory, joint positions across the reach, endpoint distributions, and joint angle measures where relevant. Variability across trials was quantified using standard deviation and covariance measures, allowing assessment of both consistency and spread of reach trajectories. For time-resolved analyses, segmented reaches were interpolated to a normalized time base so that kinematic features could be compared across trials and animals despite differences in absolute movement duration.

Standard reaching-related kinematic measures were extracted from these reconstructed trajectories. These included forelimb movement relative to the pellet, vertical and lateral trajectory extent, and endpoint position at key phases of the movement, including reach initiation, pellet approach, and terminal forelimb placement. Joint trajectories and derived measures were then used to quantify changes in reach organization, trial-to-trial variability, and group differences between control and LRN-ablated animals. Three-dimensional kinematic coordinates were reconstructed from raw two-dimensional DLC output using a custom Python analysis pipeline informed by previously described marker-tracking methods (Maghsoudi et al., 2017) and DLT calibration procedures implemented using the MATLAB software package TyDLT (Hedrick, 2008). Reconstructed three-dimensional kinematic data were processed on a trial-by-trial basis using per-trial summary files together with reconstructed coordinate files.

### Statistical analyses

For the principal endpoint and variability analyses, trials were classified by treatment group, behavioral outcome, and recording day. Treatment group was defined as control or experimental, behavioral outcome was defined as success or fail, and recording day included 12 d pre-surgery, 11 d post-surgery, 32 d post-surgery, and 39 d post-surgery. Pellet-centered coordinates were used for all analyses, including the endpoint-based analyses. For the MCP endpoint covariance plots, endpoint coordinates were taken from the peak frame associated with each reach duration result, and 95% covariance ellipses were computed to visualize group endpoint distributions.

For the derived feature analyses, per-trial kinematic features were extracted from 3D data. Trajectory variability (Fig. 5*A*) was defined as the mean Euclidean distance of an individual aligned trajectory from the condition-average trajectory after temporal resampling to a fixed number of points. Endpoint spread was defined as the Euclidean distance of each trial endpoint from the centroid of its corresponding day × group × outcome condition. Additional endpoint features included pellet-centered endpoint positions (Fig. 5*B*–*D*) in the *x*, *y*, and *z* dimensions. Overall statistical effects for these features were evaluated using linear mixed-effects models of the form value ∼ group*outcome + day + (1|rat), with rat included as a random intercept to account for repeated sampling within animals. Here group refers to the categorical predictor of experimental treatment: control (injection of the AAV2-hSyn-mCherry control construct) and experimental (LRN ablation using AAV2-mCherry-FLEX-DTA). Outcome refers to the categorical variable for success or failure. For descriptive follow-up comparisons, day-wise pairwise Welch *t*-tests were performed, and only significant comparisons were annotated in the figure panels.

For trajectory-over-time comparisons, wrist trajectories were expressed relative to the pellet and interpolated onto a common normalized time base spanning the full analysis window (*paw lift off* to reach extent), with temporal alignment referenced to the through slit event. Control and experimental groups were compared separately for each recording day in the wrist *x*, *y*, and *z* dimensions using two-tailed statistical parametric mapping (SPM; Python spm1d package) with *α* = 0.05. In this reach-level analysis, individual reaches were treated as observations. Significant suprathreshold clusters were reported for the relevant axis-by-day comparisons.

Reach duration was analyzed separately from the endpoint-feature models. For each trial, reach duration was defined from the beginning of the trial window to the frame at which the selected marker reached its minimum *x*-position within that window. Durations were computed in frames and converted to milliseconds using the frame rate of 250 frames per second. Group differences in reach duration were then evaluated independently for each recording day using two-sided Mann–Whitney *U* tests at the trial-level. These per-day duration comparisons were treated as exploratory, and significant comparisons were indicated directly on the box-plot panels.

The observational unit depended on the analysis. Wrist trajectory SPM analyses, endpoint-feature models, endpoint covariance visualizations, and reach duration comparisons used individual reaches as observations. The exploratory elbow-angle, samplewise radial-SD, and velocity-waveform analyses were first summarized within animal for each recording day, and the resulting animal-level waveforms were used for the corresponding group comparisons. Figure legends and Extended Data captions report both the number of contributing animals and the total number of reaches represented in each analysis.

Superscript letters accompanying statistical results in the Results section correspond to the statistical approaches summarized in Table 1.

**Table 1.** Statistical analyses

Letter	Data structure	Statistical test	Statistics reported
^a^	Time-normalized pellet-centered wrist trajectories; individual reaches analyzed separately by recording day and coordinate.	Two-tailed SPM independent-samples *t* test; *α* = 0.05.	Significant normalized time interval and cluster-level *p* value.
^b^	Trial-level trajectory variability, endpoint position, and endpoint spread; repeated observations grouped by rat.	Linear mixed-effects model: value ∼ group × outcome + day + (1|rat).	Coefficient (*β*), *z*/*t* statistic, and exact *p* value.
^c^	Day-wise trial-level comparisons among group-by-outcome conditions for Figure 5*A*–*E*.	Welch’s independent-samples *t* test for unequal variances.	Mean ± SEM and exact *p* value; trial counts reported by recording day and condition.
^d^	Trial-level reach duration compared between treatment groups separately by recording day.	Two-sided Mann–Whitney *U* test.	*U* statistic, exact *p* value, and group medians.

Superscript letters correspond to statistical results reported in the Results section.

### Perfusion and dissection

The precision and extent of ablation in the LRNs of our subjects was quantified using histology as follows. Rats were injected with Fatal Plus pentobarbital solution (Covetrus) at a dosage of 0.2 ml/kg as a primary euthanasia method. Rats were exsanguinated and then perfused with paraformaldehyde (PFA). Exsanguination was completed with 0.1 M phosphate buffer (PB), and perfusion was completed with 4% PFA in 0.1 M PB. The brain was cut just caudal to the pyramids to separate brainstem from spinal cord, and the entire brain was removed and placed into a vial containing 4% PFA solution in PB.

### Histology

Brains were post-fixed in 4% PFA overnight and cryoprotected in 30% sucrose for 48 h or until fully submerged. Tissue was blocked, frozen on dry ice, and cryosectioned at 30 μm. Serial sections were collected into cryoprotectant solution and stored at −20 ∘C. Sections were washed in 0.04% PBS-Tx, blocked for 1 h at room temperature in dilution buffer containing 5% neutral donkey serum, and incubated overnight at 4 ∘C with rabbit anti-dsRed primary antibody (1:1000; Living Colors DsRed Polyclonal Antibody, Takara Bio USA Inc.) in blocking buffer. Sections were then washed and incubated for 2 h at room temperature with donkey anti-rabbit Alexa Fluor 594 secondary antibody (1:1000; ThermoFisher Scientific). NeuroTrace 435/455 blue fluorescent Nissl stain was added during the final 20 min of secondary antibody incubation at a final concentration of 1:500. Sections were rinsed in PBS, mounted on Colorfrost Plus slides with Fluoromount-G, and coverslipped.

Images were collected using a Zeiss AxioImager M1 with AxioVision software. Tiled images were acquired using a 5× objective and stitched from green and red channel images. Ablation extent was assessed by comparing Nissl signal within the LRN region of experimental animals to that of control animals, while injection accuracy was evaluated from the localization of mCherry expression and the relative absence of off-target signal surrounding the LRN (Fig. 1*E*).

10.1523/ENEURO.0147-26.2026.f1-1 Figure 1-1 **RNAscope validation of vGlut1 expression within the lateral reticular nucleus supports selective FLEX-DTA targeting.** Representative coronal section through the caudal medulla showing vGlut1 labeling in red, Nissl/GFP signal in green, and DAPI nuclear counterstaining in blue. Dense bilateral vGlut1 expression is present within the lateral reticular nucleus (LRN), whereas comparatively little vGlut1 signal is visible in the immediately surrounding medullary regions. Because the experimental animals expressed Cre from the vGlut1 locus and received a Cre-dependent AAV-mCherry-FLEX-DTA construct, DTA expression was directed primarily to vGlut1-positive neurons within the LRN. This expression pattern supported selective targeting and ablation of LRN neurons while limiting DTA expression in the surrounding medullary tissue. Image acquired at 5× magnification. Download Figure 1-1, TIF file.

10.1523/ENEURO.0147-26.2026.f1-2 Figure 1-2 **RNAscope validation of minimal GABA expression within the lateral reticular nucleus supports selective FLEX-DTA targeting.** Representative coronal section through the caudal medulla showing GABA labeling in red, Nissl/GFP signal in green, and DAPI nuclear counterstaining in blue. GABA expression is comparatively sparse within the bilateral lateral reticular nucleus (LRN) relative to the surrounding medullary regions. Together with the dense vGlut1 expression observed within the LRN, this pattern supports the use of the vGlut1-Cre line and Cre-dependent AAV-mCherry-FLEX-DTA construct to direct DTA expression primarily to vGlut1-positive LRN neurons while limiting expression in surrounding tissue. Image acquired at 5× magnification. Download Figure 1-2, TIF file.

## Results

### Bilateral LRN ablation preserves the gross structure of skilled reaching

To determine how bilateral LRN ablation affected skilled reaching, we first examined whether experimental animals retained the overall spatial structure of the reach trajectory across recording days. We then asked whether more subtle abnormalities emerged in the organization and consistency of the movement by quantifying endpoint distributions, covariance, and trial-to-trial variability in pellet-centered coordinates. Finally, we assessed whether these kinematic changes were accompanied by alterations in reach timing. This stepwise approach allowed us to distinguish between gross disruption of reach generation and more selective impairments in movement precision and refinement following LRN ablation.

The initial kinematic analyses indicated that bilateral LRN ablation did not abolish the overall transport trajectory of the limb, but instead produced selective and temporally restricted alterations in wrist motion. As shown in Figure 2, pellet-centered wrist position was examined over normalized reach time in the *x*, *y*, and *z* dimensions across four recording sessions, with panels *A*–*C* corresponding to 12 d pre-surgery, panels *D*–*F* to 11 d post-surgery, panels *G*–*I* to 32 d post-surgery, and panels *J*–*L* to 39 d post-surgery. The surgical procedure involved injection of either the AAV2-hSyn-mCherry control construct or the FLEX-DTA construct, which produced LRN ablation over the subsequent time course. Across these sessions, the mean control and experimental trajectories generally followed similar time courses, and the overall shape of the wrist trajectory was largely preserved between groups despite the emergence of axis-specific differences during discrete portions of the reach (Fig. 2*A*–*L*).

10.1523/ENEURO.0147-26.2026.f2-1 Figure 2-1 **Three-dimensional elbow-angle waveforms across normalized reach time.** Three-dimensional elbow angle was calculated as the included angle between the shoulder-to-elbow and wrist-to-elbow vectors and expressed across a normalized reach interval from 0% to 100%. Panels show elbow-angle waveforms at 12 days pre-surgery, 11 days post-surgery, 32 days post-surgery, and 39 days post-surgery. Thin gray curves represent the mean elbow-angle waveform for each individual animal, calculated by averaging all analyzed reaches from that animal at the corresponding recording day. The thick solid blue and dashed orange curves represent the control and experimental group means, respectively. Shaded regions indicate the group mean ± SEM across animal-level mean waveforms and therefore represent between-animal uncertainty rather than trial-level variability. At 12 days pre-surgery, control animals contributed 13 reaches from 2 rats and experimental animals contributed 44 reaches from 3 rats. At 11 days post-surgery, control animals contributed 12 reaches from 2 rats and experimental animals contributed 26 reaches from 3 rats. At 32 days post-surgery, control animals contributed 14 reaches from 2 rats and experimental animals contributed 12 reaches from 3 rats. At 39 days post-surgery, control animals contributed 8 reaches from 2 rats and experimental animals contributed 28 reaches from 3 rats. Independent two-sample SPM{*t*} analyses were performed on the animal-level mean elbow-angle waveforms separately at each recording day using α = 0.05. No significant control–experimental differences or significant suprathreshold clusters were detected across normalized reach time at any recording day. Accordingly, visible separation between group means at portions of the waveform should be interpreted as descriptive rather than statistically significant. Download Figure 2-1, TIF file.

Reach-level SPM (*α* = 0.05) further supported this interpretation, identifying significant group differences only in a limited subset of comparisons: a brief cluster in the *x*-dimension on the pre-surgical session in panel *A* at normalized time 0.0000–0.0206 (*p* = 0.0483), a second cluster in the *x*-dimension at 32 d post-surgery in panel *G* at normalized time 0.3076–0.3439 (*p* = 0.0435), and a broader cluster in the *z*-dimension at 39 d post-surgery in panel *L* at normalized time 0.1342–0.2662 (*p* = 0.0028); all remaining axis-wise comparisons were non-significant. Because the *x*-dimension difference in panel *A* was present before ablation, it was interpreted cautiously and was not considered by itself to indicate a lesion-induced effect. This pre-surgical difference likely reflects animal-to-animal variation in initial body or forelimb positioning within the reaching chamber before reach initiation. Although pellet-centered coordinates controlled for target location, individual animals could still begin reaches from slightly different postural positions relative to the slit and pellet, including being positioned slightly farther forward or farther back. Therefore, baseline offsets in the early portion of the reach were treated as pre-existing positional variability rather than evidence of an LRN-dependent effect. This pattern argues against a generalized failure of reach execution following LRN ablation. Rather than disrupting wrist transport throughout the entire movement, the lesion effect appeared to emerge only during restricted temporal intervals and in specific spatial dimensions, consistent with a more selective disturbance of movement control.

**Figure 3.**
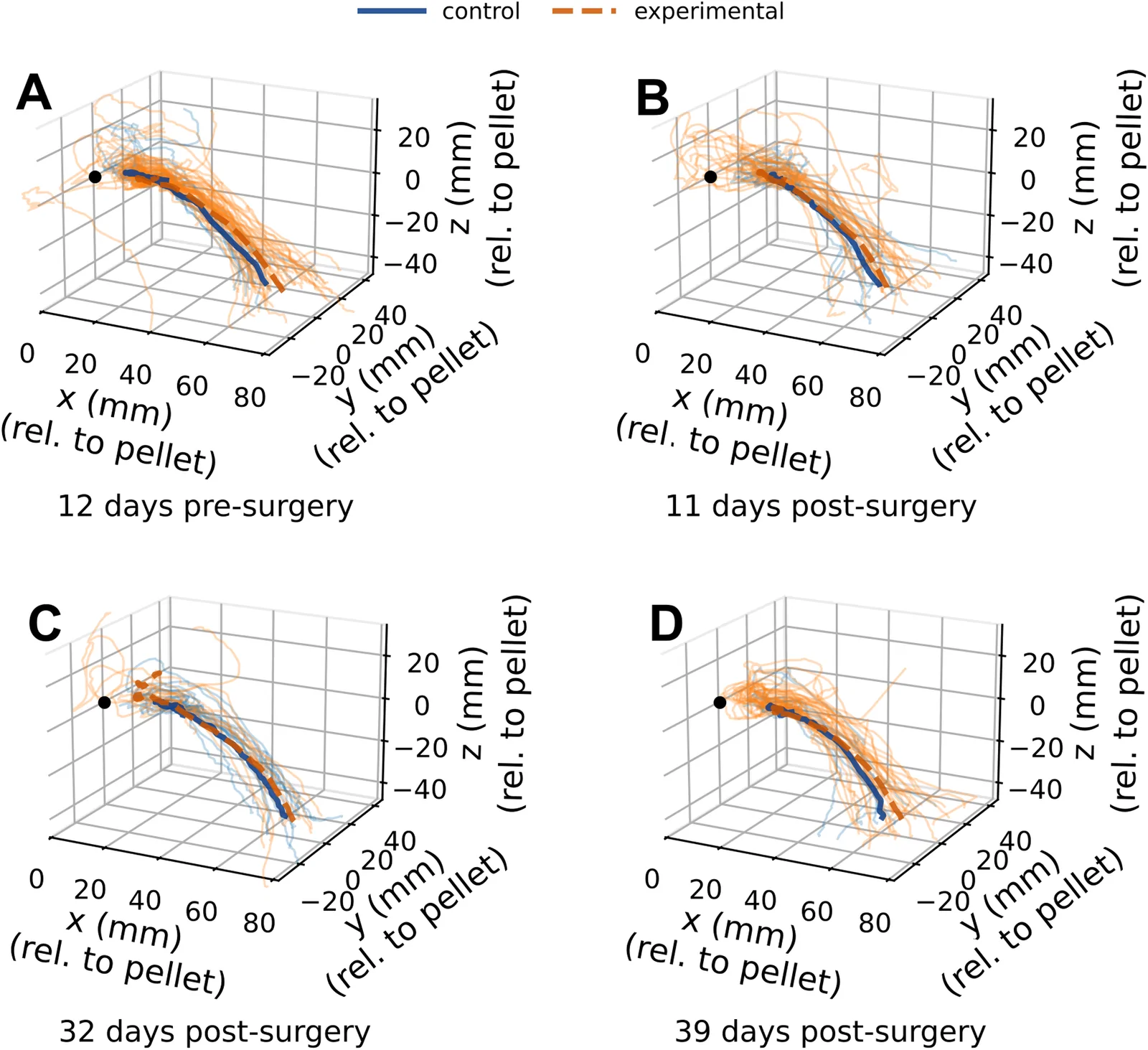
Comparison of Control versus Experimental wrist 3D trajectories. Panels ***A***–***D***, correspond to day −12, day +11, day +32, and day +39, respectively. Wrist trajectories are shown in pellet-centered coordinates with thin lines denoting individual trials and thick lines denoting group mean trajectories for control and experimental animals. Across days, both groups followed similar gross reach paths, with visible day-specific differences in mean trajectory shape and trial-to-trial dispersion. This figure provides a spatial visualization of wrist trajectories; formal axis-wise statistical comparisons over normalized reach time are reported in Figure 2. The black point marks the pellet origin. Endpoint variability was notably larger in experimental animals at the +39 time point, seen here as dispersion of individual orange lines. Corresponding samplewise within animal variability of pellet-centered MCP trajectories is provided in Extended Data Figure 3-1.

To determine whether these trajectory findings were accompanied by changes in proximal joint kinematics, we also performed an exploratory three-dimensional elbow-angle analysis. Elbow-angle waveforms were averaged within animal and compared between control and experimental groups across normalized reach time (Extended Data Fig. 2-1). No significant control—experimental differences or suprathreshold SPM clusters were detected at any recording day. The elbow angle at reach onset was also not significantly different between groups. Thus, within the resolution and statistical power of the present dataset, the endpoint deficits were not accompanied by a detectable group-level alteration in elbow-angle trajectory. This does not exclude differences in shoulder configuration, trunk posture, wrist orientation, or coordination across multiple joints, any of which could alter endpoint position while elbow-angle remains similar. Figure 3 reinforces this conclusion from a spatial perspective. In panels *A*–*D*, which correspond to the same dates as in Figure 2, pellet-centered wrist trajectories from control and experimental animals followed broadly similar gross paths toward the pellet origin, with thin lines representing individual trials and thick lines representing group mean trajectories. Although visible day-specific differences in mean trajectory shape and trial-to-trial dispersion were present, the overall 3D organization of the reach remained recognizable in the experimental group across all days (Fig. 3). Taken together, Figures 2 and 3 suggest that bilateral LRN ablation left the task of skilled reaching largely intact while introducing modest but statistically detectable deviations in selected dimensions and phases of the movement. This distinction is important for interpretation of the subsequent analyses, because it indicates that the principal effect of LRN ablation is unlikely to reflect simple loss of reach generation, but instead points toward a more specific impairment in the refinement, consistency, and fine spatial control of the pellet-directed forelimb trajectory. To examine trajectory variability continuously across the reach, we also calculated the three-dimensional radial standard deviation of the pellet-centered MCP trajectory at each normalized timepoint within each animal (Extended Data Fig. 3-1). The animal-level curves showed descriptive differences in the magnitude and timing of trajectory variability, but independent SPM analyses detected no significant control–experimental clusters at any recording day. These results provide a complementary description of within animal trajectory variability without indicating a significant waveform-level group difference.

10.1523/ENEURO.0147-26.2026.f3-1 Figure 3-1 **Samplewise within-animal variability of pellet-centered MCP trajectories.** Samplewise trajectory variability was quantified as the three-dimensional radial standard deviation of the pellet-centered metacarpophalangeal joint (MCP) trajectory at each normalized reach timepoint. Trial trajectories were expressed relative to the pellet position and interpolated from 0% to 100% of the analyzed reach interval. Panels show radial-SD waveforms at 12 days pre-surgery, 11 days post-surgery, 32 days post-surgery, and 39 days post-surgery. Thin blue and orange curves represent the radial-SD waveform calculated separately for each contributing control or experimental animal from that animal’s repeated reaches. Thick solid blue and dashed orange curves represent the means of the control and experimental animal-level radial-SD waveforms, respectively. The shaded regions represent the group mean ± standard error of the mean (SEM) across animals. These shaded bands are not error flags, confidence limits for individual reaches, or percentile ranges. Rather, at each normalized reach timepoint, the SEM describes the uncertainty in the estimated group mean caused by variation among the contributing animal-level radial-SD curves. A narrower shaded band indicates that animals in that group produced more similar radial-SD values at that timepoint, whereas a wider band indicates greater between-animal variation and therefore less precision in the estimated group mean. Because SEM depends on both the between-animal standard deviation and the number of contributing animals, the bands should be interpreted cautiously in this small cohort and should not be used as a direct measure of the total variability among all reaches. Animals were required to contribute at least two reaches within a recording day to generate a within-animal radial-SD waveform. At 12 days pre-surgery, the control group included 2 animals and 13 reaches, and the experimental group included 3 animals and 44 reaches. At 11 days post-surgery, the control group included 2 animals and 12 reaches, and the experimental group included 3 animals and 26 reaches. At 32 days post-surgery, the control group included 2 animals and 14 reaches, whereas the experimental within-animal summary included 2 animals and 11 reaches; a third experimental animal contributed one reach that remained included in the complete pooled dataset but could not independently generate an SD waveform. At 39 days post-surgery, the control group included 2 animals and 8 reaches, and the experimental group included 3 animals and 28 reaches. Independent two-sample statistical parametric mapping tests, SPM{*t*}, were performed on the animal-level radial-SD waveforms separately at each recording day using α = 0.05. No significant control–experimental differences or suprathreshold clusters were detected at any recording day. Download Figure 3-1, TIF file.

### LRN ablation increases endpoint dispersion without eliminating pellet-directed reaching

**Figure 4.**
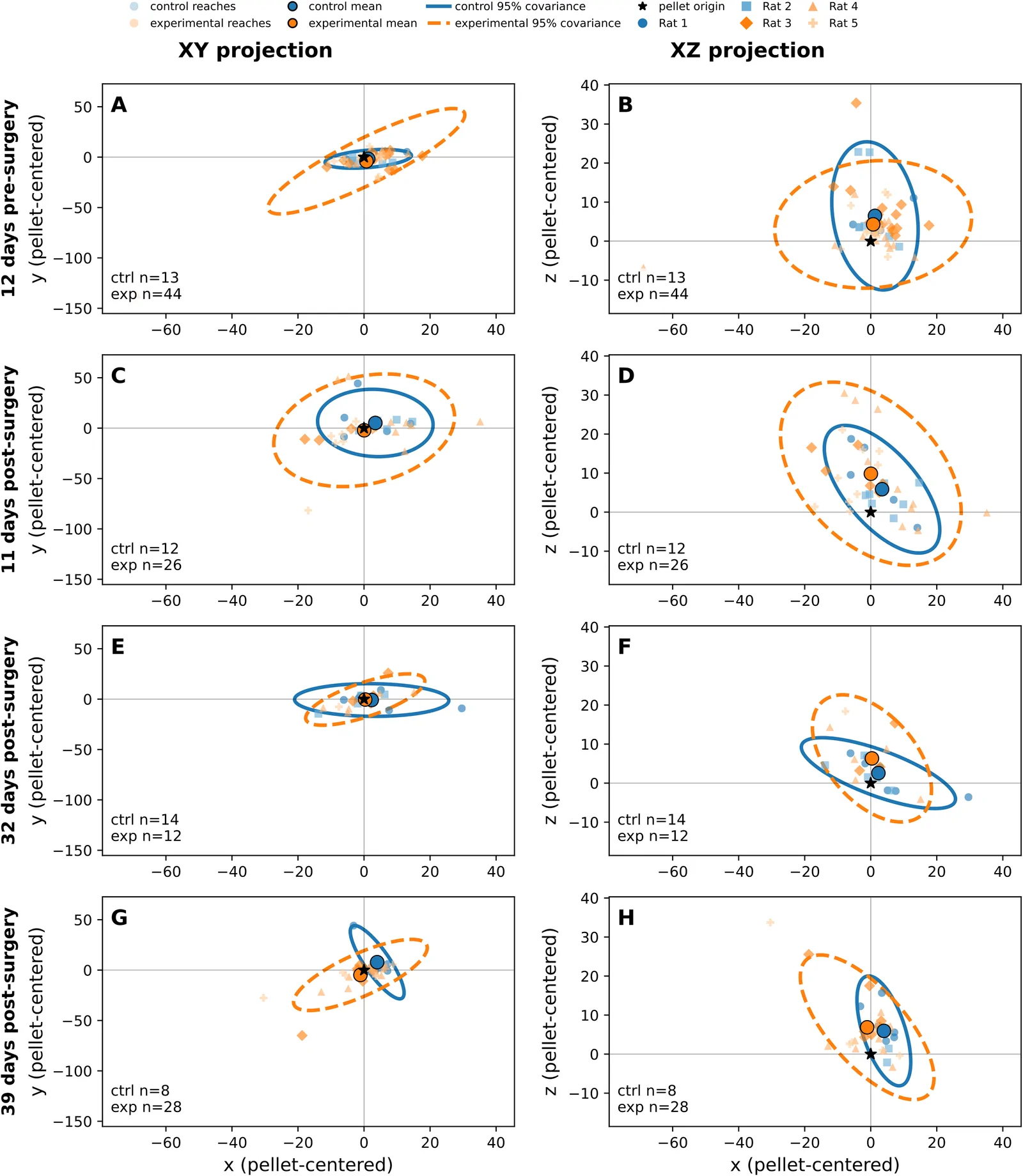
Pellet-centered MCP endpoint covariance in control and experimental animals across recording days. Panels ***A***, ***C***, ***E***, and ***G***, show the pellet-centered *x*–*y* projection of MCP endpoints, and panels ***B***, ***D***, ***F***, and ***H***, show the corresponding *x*–*z* projection. Rows correspond to four recording days: ***A***, ***B***, 12 d pre-surgery; ***C***, ***D***, 11 d post-surgery; ***E***, ***F***, 32 d post-surgery; and ***G***, ***H***, 39 d post-surgery. Small points represent individual reaches, large filled circles represent group mean endpoints, ellipses represent the 95% covariance regions, and the black star marks the pellet origin. Different shades of blue and orange identify individual animals, as indicated in the legend. Corresponding bagplots showing depth-based endpoint distributions, peripheral observations, and animal identity are provided in Extended Data Figure 4-1.

Subsequent analyses indicated that the more prominent consequences of bilateral LRN ablation emerged not in the gross transport path of the limb, but in the spatial organization and consistency of pellet-directed endpoints. This pattern is illustrated in Figure 4, which shows pellet-centered MCP endpoint distributions across recording days in both the *x*–*y* projection and the corresponding *x*–*z* projection. As a complementary distribution-sensitive visualization, Extended Data Figure 4-1 presents bagplots of the same endpoint populations, with symbol shape identifying the individual animal contributing each reach. The bags enclose the central approximately 50% of each bivariate endpoint distribution, while the surrounding fences provide a robust representation of the broader distribution. The *x*–*y* projection provides a top-down view of paw placement relative to the pellet, with positive *x* corresponding to movement toward the pellet through the slit, consistent with the axis convention shown in Figure 2*J*–*L*. The *x*–*z* projection shows the corresponding fore-aft and vertical organization of the endpoint distribution.

10.1523/ENEURO.0147-26.2026.f4-1 Figure 4-1 **Bagplots of pellet-centered MCP endpoint distributions in control and experimental animals.** Panels **A, C, E,** and **G** show the pellet-centered x–y projection of metacarpophalangeal joint (MCP) endpoints, and panels **B, D, F,** and **H** show the corresponding x–z projection. Rows correspond to 12 days pre-surgery (**A, B**), 11 days post-surgery (**C, D**), 32 days post-surgery (**E, F**), and 39 days post-surgery (**G, H**). Each plotted symbol represents the endpoint of one reach. Blue and orange symbols denote control and experimental reaches, respectively, and symbol shape identifies the individual animal contributing each observation. The filled blue and orange circles indicate the control and experimental multivariate depth medians. The shaded blue and orange bags enclose the central approximately 50% of each endpoint distribution according to Tukey data depth. The surrounding loop or fence is derived by expanding the bag relative to the depth median and provides a robust representation of the broader bivariate distribution. Points outside the fence remain displayed and represent peripheral observations rather than automatically excluded data. The black star marks the pellet origin. Trial counts were control *n* = 13 and experimental *n* = 44 at 12 days pre-surgery; control *n* = 12 and experimental *n* = 26 at 11 days post-surgery; control *n* = 14 and experimental *n* = 12 at 32 days post-surgery; and control *n* = 8 and experimental *n* = 28 at 39 days post-surgery. Bagplots are presented as descriptive, distribution-sensitive complements to the covariance ellipses and were not used as a separate inferential test. Download Figure 4-1, TIF file.

**Figure 5.**
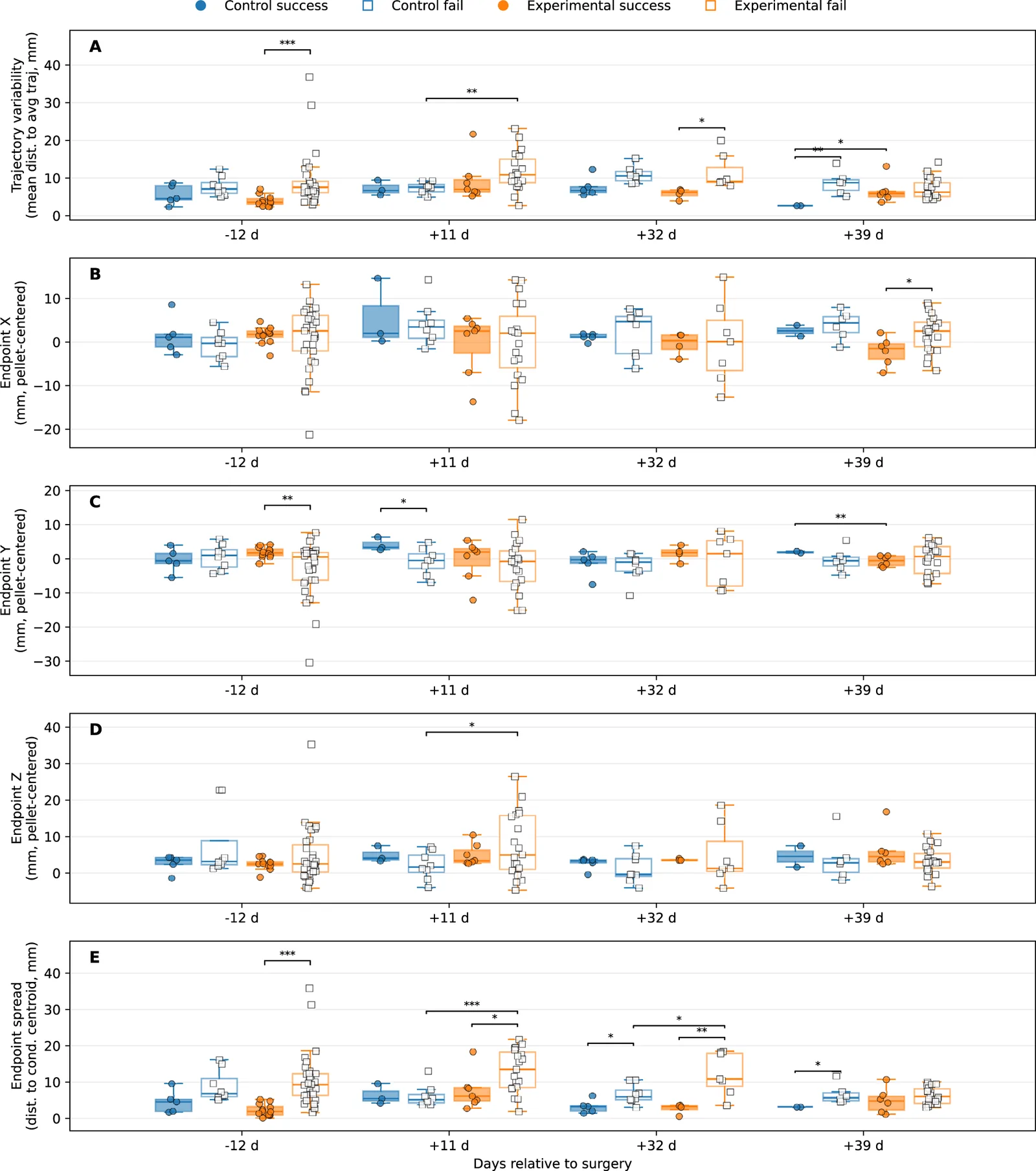
Compiled feature analysis of reaches. ***A***, Trajectory variability, calculated as mean Euclidean distance from the condition-average aligned trajectory. ***B***, Pellet-centered MCP endpoint position in the *X* dimension. ***C***, Pellet-centered MCP endpoint position in the *Y* dimension. ***D***, Pellet-centered MCP endpoint position in the *Z* dimension. ***E***, Endpoint spread, calculated as the Euclidean distance of each trial endpoint from the day×group×outcome condition centroid. For each recording day, box-and-whisker plots with overlaid trial points show control success, control fail, experimental success, and experimental fail trials. Day-wise brackets indicate significant descriptive Welch unequal-variance comparisons. Numerical values reported in the Results are condition means ± standard error of the mean (SEM); the boxes display the median and interquartile range of the trial-level distributions.

Across sessions, the experimental group frequently exhibited broader 95% covariance ellipses and mean endpoints that appeared more displaced relative to both the pellet origin and the corresponding control distributions. On the pre-surgical session, endpoint spread was already somewhat greater in the experimental group, although both groups remained relatively constrained in space. This indicates that some differences in endpoint position and dispersion preceded LRN ablation and were therefore not interpreted as surgery-induced effects. Similar to the early trajectory differences described above, these baseline differences may reflect natural animal-to-animal variation in initial body posture or forelimb position relative to the slit and pellet, despite the use of pellet-centered coordinates. At 11 d post-surgery, the endpoint cloud in the experimental group remained visibly broader than that of controls, suggesting reduced trial-to-trial consistency in where reaches terminated relative to the pellet (Fig. 4*C*,*D*). This impression persisted at 32 d post-surgery, despite the smaller number of included trials on that day, and was especially pronounced at 39 d post-surgery, where experimental distributions appeared both more scattered and less tightly centered, including visibly larger spread along the *x* dimension. Because all endpoints were plotted in pellet-centered coordinates, this broader and more displaced covariance has a direct functional interpretation: the limb continued to approach the target, but the final placement of the distal forelimb relative to the pellet was less spatially constrained after LRN ablation. Thus, Figure 4 suggests that the principal lesion-related abnormality was not simple failure to launch the reach, but rather diminished precision and reproducibility of the movement as it approached its endpoint. Quantitative analyses of pellet-centered endpoint position and endpoint spread, which provide statistical support for the distributional patterns visualized in Figure 4, are presented in Figure 5.

### Quantitative feature analysis identifies endpoint spread as the clearest lesion-related deficit

Figure 5 provides quantitative support for the endpoint position and dispersion patterns visualized in Figure 4 and indicates that bilateral LRN ablation preferentially disrupted movement consistency and endpoint precision rather than the overall execution of the reach. For each recording day, box-and-whisker plots with overlaid trial points show control success, control fail, experimental success, and experimental fail trials. Overall statistics were assessed using linear mixed-effects models of the form value ∼ group × outcome + day + (1|rat), with rat included as a random intercept to account for repeated observations within animals. Trial counts were as follows: on day −12, control fail (*n* = 8), control success (*n* = 5), experimental fail (*n* = 32), and experimental success (*n* = 12); on day +11, control fail (*n* = 9), control success (*n* = 3), experimental fail (*n* = 19), and experimental success (*n* = 7); on day +32, control fail (*n* = 9), control success (*n* = 5), experimental fail (*n* = 7), and experimental success (*n* = 5); and on day +39, control fail (*n* = 6), control success (*n* = 2), experimental fail (*n* = 22), and experimental success (*n* = 6).

Among the features examined, the clearest overall lesion-related effect was observed for endpoint spread (panel *E*), defined as the Euclidean distance of each trial endpoint from its day×group×outcome condition centroid. Mixed-effects modeling revealed a significant main effect of group for this measure (*β* = 3.2960, *z*/*t* = 3.1186, *p* = 0.0018^*b*^), indicating that, across recording days and outcomes, experimental animals exhibited broader endpoint dispersion than controls. By contrast, the outcome term (*p* = 0.0662^*b*^) and the group × outcome interaction (*p* = 0.0705^*b*^) did not reach significance at the model level, although both trended toward an effect, suggesting that endpoint variability may also have been shaped in part by behavioral success. Day-wise descriptive Welch comparisons were consistent with this interpretation. Within the experimental group, failed reaches showed substantially greater endpoint spread than successful reaches on the pre-surgical day [10.639 ± 1.347 vs 2.358 ± 0.476 mm, *p* = (1.24 × 10^−6^)^*c*^], at 11 d post-surgery (13.205 ± 1.356 vs 7.719 ± 1.947 mm, *p* = 0.0389^*c*^), and at 32 d post-surgery (12.420 ± 2.236 vs 2.761 ± 0.709 mm, *p* = 0.0043^*c*^). Cross-group comparisons of failed reaches were also significant at 11 d post-surgery (13.205 ± 1.356 vs 6.242 ± 1.101 mm, *p* = 0.0006^*c*^), indicating that failed reaches in the experimental group were especially spatially dispersed relative to controls. At 32 d post-surgery, control failed reaches showed greater endpoint spread than control successful reaches (6.706 ± 0.951 vs 3.364 ± 0.828 mm, *p* = 0.0229^*c*^), and experimental failed reaches showed greater endpoint spread than control failed reaches (12.420 ± 2.236 vs 6.706 ± 0.951 mm, *p* = 0.0461^*c*^). These findings suggest that the endpoint consequences of LRN ablation were expressed most strongly as reduced clustering of pellet-directed forelimb placement, particularly under conditions in which reaches were unsuccessful.

A similar, though somewhat weaker, pattern was observed for trajectory variability (Fig. 5*A*), which quantified the mean Euclidean deviation of each aligned reach from its condition-average trajectory. At the model level, neither group (*p* = 0.1200^*b*^) nor outcome (*p* = 0.0755^*b*^) nor their interaction (*p* = 0.4925^*b*^) reached significance, indicating that trajectory-wide variability did not differ uniformly across all conditions. Nevertheless, the day-wise comparisons showed that failed reaches were often less stereotyped than successful reaches, particularly within the experimental group. On the pre-surgical day, experimental failures exhibited greater variability than experimental successes (9.301 ± 1.280 vs 3.924 ± 0.420 mm, *p* = 0.0003^*c*^); the same pattern was present at 32 d post-surgery (11.486 ± 1.732 vs 5.785 ± 0.656 mm, *p* = 0.0163^*c*^). At 11 d post-surgery, experimental failed reaches were more variable than control failed reaches (11.756 ± 1.191 vs 7.382 ± 0.503 mm, *p* = 0.0025^*c*^), and also more variable than control successful reaches (11.756 ± 1.191 vs 7.214 ± 1.175 mm, *p* = 0.0286^*c*^). Together, these comparisons suggest that although trajectory variability in Figure 5*A* did not show a uniform global lesion effect, trajectory consistency was reduced in specific day- and outcome-dependent contexts. Additional later-session abnormalities were detected in other measures, including the wrist *z* trajectory at 39 d post-surgery in Figure 2*L* and endpoint position comparisons at 39 d post-surgery in Figure 5*B*, *C*. Collectively, these findings point toward instability and selective alteration of reach execution rather than loss of the gross movement.

Analyses of pellet-centered endpoint position (Fig. 5*B*–*D*) in the individual spatial dimensions further indicated that lesion-related abnormalities were not distributed uniformly across coordinates. For endpoint *x* (panel *B*), the overall mixed model revealed no significant main effects or interaction (group: *p* = 0.3351^*b*^; outcome: *p* = 0.9258^*b*^; group × outcome: *p* = 0.5487^*b*^), yet day-wise comparisons identified significant differences at 39 d post-surgery. Experimental successful reaches terminated at a more negative *x*-position than experimental failed reaches (−2.097 ± 1.334 vs 1.957 ± 0.902 mm, *p* = 0.0302^*c*^), and also differed from control failed reaches (−2.097 ± 1.334 vs 3.903 ± 1.331 mm, *p* = 0.0097^*c*^). Endpoint *y* (panel *C*) showed a comparable absence of effects (group: *p* = 0.4008^*b*^; outcome: *p* = 0.4060^*b*^; interaction: *p* = 0.6414^*b*^), but yielded several notable day-wise differences, including a difference between successful and failed reaches in the experimental group on the pre-surgical day, and a cross-group difference among successful reaches at 39 d post-surgery, where experimental successful reaches terminated at a lower *y*-position than control successful reaches (−0.638 ± 0.626 vs 1.978 ± 0.266 mm, *p* = 0.0085^*c*^). Endpoint *z* (panel *D*) similarly showed no significant effects (all *p* ≥ 0.3941^*b*^), with the only significant day-wise difference occurring among failed reaches at 11 d post-surgery, where experimental animals terminated farther from the pellet in *z* than controls (7.784 ± 2.038 vs 2.127 ± 1.361 mm, *p* = 0.0296^*c*^). Taken together, these results indicate that bilateral LRN ablation did not impose a simple, fixed spatial offset on reach endpoints. Instead, the strongest and most consistent abnormality was an increase in endpoint dispersion, accompanied by selective day- and outcome-dependent shifts in specific endpoint coordinates. Therefore, these findings do support the interpretation that the LRN contributes less to the overall production of the reach trajectory than to the trial-by-trial stabilization and refinement of pellet-directed forelimb placement.

### Reach duration differences emerge later than endpoint precision deficits

**Figure 6.**
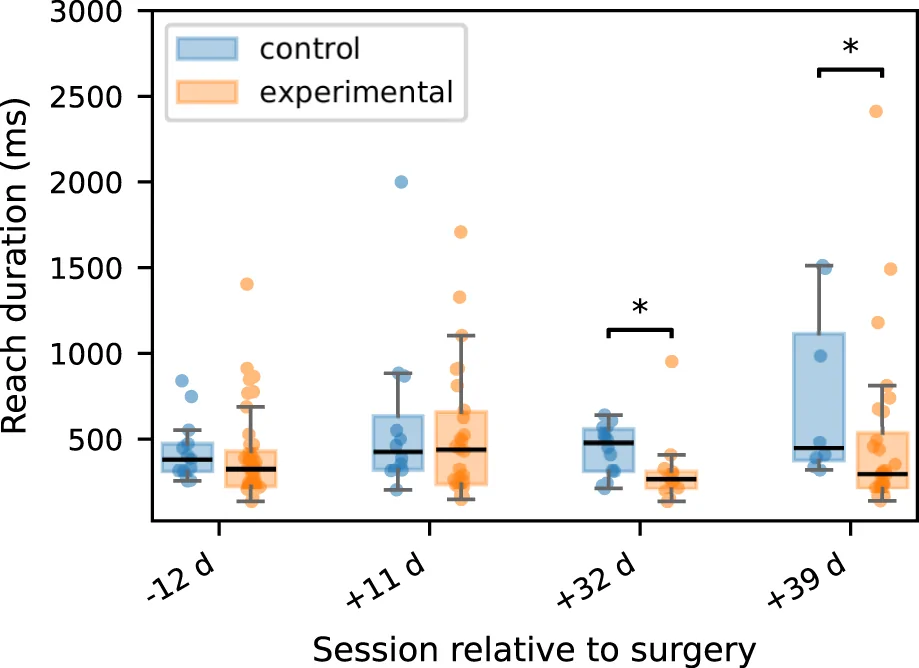
Reach duration across recording day and treatment group. Box-plots show trial-level reach duration for control and experimental animals across recording days, with individual trials overlaid. Reach duration distributions were similar between groups on day −12 and day +11, whereas exploratory day-wise comparisons indicated shorter reach durations in experimental animals on day +32 and day +39. Asterisks indicate two-sided Mann–Whitney *U* tests performed separately for each day at the trial-level; significant differences were observed on +32 (*U* = 128.0, *p* = 0.025^*d*^) and +39 (*U* = 169.0, *p* = 0.031^*d*^). Corresponding animal-level three-dimensional velocity waveforms and SPM comparisons are provided in Extended Data Figure 6-1.

The reach duration analysis indicated that temporal changes after bilateral LRN ablation were present, but were more limited and less consistent than the spatial differences identified in the preceding endpoint analyses (Figs. 4, 5). As shown in Figure 6, reach duration distributions overlapped substantially between control and experimental animals on the pre-surgical session and at 11 d post-surgery, with no significant per-day treatment differences detected at either time point (12 d pre-surgery: *U* = 364.5, *p* = 0.138^*d*^; 11 d post-surgery: *U* = 177.0, *p* = 0.520^*d*^). Descriptive statistics were consistent with this. On the pre-surgical day, median reach duration was 384 ms in controls and 326 ms in experimental animals, while at 11 d post-surgery the medians were 426 ms and 438 ms, respectively. By contrast, significant group differences emerged at the later post-surgical sessions. At 32 d post-surgery, experimental animals exhibited shorter reach durations than controls (*U* = 128.0, *p* = 0.0253^*d*^), with median values of 266 ms in the experimental group versus 476 ms in controls. A similar pattern was present at 39 d post-surgery (*U* = 169.0, *p* = 0.0315^*d*^), where median reach duration was 298 ms in experimental animals compared with 448 ms in controls. Thus, unlike the earlier trajectory analyses in Figures 2 and 3, which showed broadly preserved reach trajectory and structure with only temporally focused differences, Figure 6 suggests that timing changes were not an immediate result of LRN ablation, but instead emerged later in the post-surgical period. To examine how velocity was organized across the movement, we also calculated three-dimensional velocity waveforms across normalized reach time (Extended Data Fig. 6-1). Although visible differences in the group mean profiles were present on some recording days, animal-level SPM analyses detected no significant control–experimental clusters at any recording day. The velocity waveforms are therefore presented as descriptive context for the reach duration findings rather than as evidence of an independent significant waveform effect.

10.1523/ENEURO.0147-26.2026.f6-1 Figure 6-1 **Three-dimensional velocity waveforms across normalized reach time.** Three-dimensional forelimb velocity was calculated from the reconstructed reach trajectories and expressed in millimeters per second across a common normalized time base from 0% to 100% of the analyzed reach interval. Panels show velocity waveforms at 12 days pre-surgery, 11 days post-surgery, 32 days post-surgery, and 39 days post-surgery. Thin gray curves represent individual rat-level mean velocity waveforms. The thick solid blue and dashed orange curves represent the control and experimental group means, respectively, and the shaded regions indicate mean ± standard error of the mean (SEM) across the contributing animal-level waveforms. Panel annotations report the number of contributing animals and the total number of reaches represented in each group. At 12 days pre-surgery, control animals contributed 13 reaches from 2 rats and experimental animals contributed 44 reaches from 3 rats. At 11 days post-surgery, control animals contributed 12 reaches from 2 rats and experimental animals contributed 26 reaches from 3 rats. At 32 days post-surgery, control animals contributed 14 reaches from 2 rats and experimental animals contributed 12 reaches from 3 rats. At 39 days post-surgery, control animals contributed 8 reaches from 2 rats and experimental animals contributed 28 reaches from 3 rats. Independent two-sample SPM{*t*} analyses were performed on rat-level mean velocity waveforms separately at each recording day using α = 0.05. No significant control–experimental differences or suprathreshold clusters were detected at any recording day. Therefore, visible group separations in the velocity waveforms should be interpreted descriptively. Download Figure 6-1, TIF file.

## Discussion

The present findings indicate that bilateral LRN ablation altered skilled forelimb behavior primarily by degrading the precision and stability of pellet-directed reaching rather than by abolishing the basic transport pattern of the movement. In the trajectory-based analyses, experimental animals retained broadly recognizable wrist paths toward the pellet in both the axis-by-time plots shown in Figure 2 and the 3D trajectory reconstructions shown in Figure 3, with statistically significant group differences limited to a small number of temporally restricted SPM clusters in wrist *x* on day −12 (Fig. 2*A*) and day +32 (Fig. 2*G*), and in wrist *z* on day +39 (Fig. 2*L*); all remaining axis-wise comparisons were non-significant. This relative preservation of the gross kinematic trajectory contrasted with the endpoint-level dispersion visualized in Figure 4 and quantified in Figure 5, where experimental animals showed a wider range of MCP endpoint covariance, increased endpoint spread, and greater context-dependent variability, indicating that the final placement of the forelimb relative to the pellet was less spatially constrained after LRN loss. In particular, Figure 4 showed broader and more displaced endpoint covariance in the experimental group across recording days, while Figure 5 identified endpoint spread as the clearest overall lesion-related effect. Reach duration, shown in Figure 6, was also altered, but only at the later post-surgical sessions, where experimental animals exhibited shorter reaches than controls at day +32 and day +39, suggesting that temporal differences emerged after, and were less dominant than, the spatial abnormalities. Taken together, these findings support the central interpretation that the LRN contributes less to the generation of the reach in an all-or-none sense than to the trial-by-trial assessment and stabilization of pellet-directed forelimb movement.

The difference between overall trajectory and variability between endpoint organization is informative for interpreting the functional contribution of the LRN. As shown in Figures 2 and 3, experimental animals continued to generate a broadly recognizable pellet-directed wrist trajectory, and formal axis-wise comparisons revealed only a small number of temporally restricted SPM differences rather than a total disruption of the entire reach. By contrast, Figure 4 visualized, and Figure 5 quantitatively evaluated, the more robust lesion-related phenotype at the endpoint level, where experimental animals displayed broader MCP endpoint covariance and significantly greater endpoint spread than controls. In the mixed-effects analysis of endpoint spread, experimental versus control (group) was the clearest overall predictor, with experimental reaches exhibiting greater dispersion across recording days and outcomes, whereas the effects of outcome and the group-by-outcome interaction were weaker and did not reach conventional significance thresholds in the MCP analysis. This pattern argues against the interpretation that ablation of the LRN simply caused a fixed spatial offset or a nonspecific loss of movement (reach generation). Instead, it suggests that the basic reaching trajectory remained intact, while the trial-by-trial regulation of forelimb placement relative to the pellet became less stable. Functionally, this difference is more consistent with the role of the LRN being primarily involved in refinement, repetition, or adjustment of skilled reaching than in the initiation of the reaching task itself. Thus, the present data suggests that even when the limb could still move toward the target along a general path, the absence of normal LRN contribution was associated with reduced precision in how that movement ultimately brought the forelimb to the pellet.

An additional point emerging from the endpoint analyses is that the effect of bilateral LRN ablation was not expressed exclusively as a shift in group means, but was shaped by trial outcome and by specific kinematic features. In Figure 5, failed reaches were often associated with broader distributions and larger values for both the trajectory variability (Fig. 5*A*) and the endpoint spread, most particularly within the experimental group, and thereby indicating that the lesion-related disruption became most apparent under conditions in which the behavior was already less successful. For endpoint spread, for example, failed experimental reaches were substantially more dispersed than successful experimental reaches on the pre-surgical session, at 11 d post-surgery, and again at 32 d post-surgery. Experimental failed reaches also showed greater endpoint spread than control failed reaches at 11 and 32 d post-surgery. At 32 d post-surgery, failed reaches were additionally more dispersed than successful reaches within the control group. A similar pattern was present for trajectory variability (Fig. 5*A*), where failed reaches in the experimental group frequently showed less stereotyped behavior in their trajectories than successful reaches. This information suggests that LRN ablation did not simply impose a single fixed deficit on all reaches. Instead, some reaches remained relatively well organized, whereas others deviated more markedly in endpoint precision or trajectory consistency, furthering the hypothesis that LRN ablation does not simply impose a single fixed deficit on all reaches.

This interpretation is strengthened by the fact that the strongest and most consistent difference across endpoint analyses was within increased dispersion. In Figure 5, the mixed-effects model for endpoint *x*, *y*, and *z* did not reveal the same group effect seen for endpoint spread, and the significant day-wise comparisons in individual coordinates were selective. This pattern argues against a simple directional bias in which the LRN ablation caused the forelimb to terminate consistently too far in one direction. Instead, the data are more consistent with reduced repetitive trajectory (i.e., the forelimb trajectory arrived at a broader range of positions relative to the pellet from trial-to-trial). This outcome-dependent increase in variability suggests that the LRN normally contributes to the reliability with which skilled forelimb trajectories are refined into stable, reproducible pellet-directed endpoints.

One limitation of the endpoint analysis is that endpoint spread was already somewhat greater in the experimental group during the pre-surgical session. Animals were not assigned to control or ablation groups based on pre-surgical endpoint covariance or endpoint spread, and therefore this baseline difference is interpreted cautiously. We do not attribute the pre-surgical difference to LRN loss. Instead, the interpretation of reduced endpoint precision is based on the persistence and later prominence of endpoint dispersion after surgery, together with the mixed-effects model showing a significant group effect for endpoint spread. This pattern suggests that LRN ablation was associated with reduced endpoint stabilization rather than a simple fixed spatial offset. Although the most prominent abnormalities after bilateral LRN ablation were spatial rather than temporal, the reach duration analysis suggests that the temporal organization of pellet-directed behavior was also altered at later post-surgical stages. Reach duration distributions overlapped substantially between control and experimental animals on the pre-surgical session and at 11 d post-surgery, whereas differences emerged at the later post-surgical sessions, where experimental animals exhibited shorter reaches than controls. This was most apparent at day +32 and day +39, when experimental reaches were significantly shorter than control reaches (Fig. 6). The delayed emergence of these temporal effects may reflect the time course of DTA-mediated neuronal loss rather than an immediate behavioral consequence of surgery itself. Diphtheria toxin subunit A acts by inhibiting protein synthesis in targeted cells, and viral expression followed by toxin-mediated cellular degeneration is expected to unfold over days to weeks rather than instantaneously (Uchida, 2014; Rentsch et al., 2019). In the present study, the lack of a significant reach duration difference at 11 d post-surgery, followed by significant differences at 32 and 39 d post-surgery, is therefore consistent with a progressive lesion effect becoming more behaviorally apparent as LRN neuronal loss matured. This interpretation should be viewed cautiously, however, because lesion progression was not measured histologically at each behavioral time point. One possible interpretation is that slower reaches in control animals may preserve more time for online adjustment of the forelimb trajectory as the paw approaches the pellet. The normalized velocity profiles provide descriptive context for where these timing differences may arise within the reach, although no significant animal-level velocity-waveform clusters were detected (Extended Data Fig. 6-1). In this context, a longer reach duration may not simply reflect slower movement, but may instead allow additional opportunity for corrective refinement, improving the likelihood that the distal forelimb arrives at a precise and reproducible endpoint. By contrast, the shorter reach duration observed in experimental animals may reflect a more rapid or ballistic execution strategy, in which the limb is still directed toward the pellet but with less time available for trajectory adjustment before endpoint placement. This interpretation is consistent with the increased endpoint dispersion and greater trial-to-trial variability observed in the experimental group. Thus, the shorter reaches observed at later post-surgical stages are unlikely to reflect improved motor efficiency, but rather may indicate altered temporal control of skilled reaching after LRN ablation.

In conclusion, bilateral LRN ablation did not eliminate the ability of a rodent to generate pellet-directed reaches, but instead altered the quality with which those movements were executed. The most consistent abnormalities were observed not in the gross transport trajectory of the limb, but in the precision, reproducibility, and spatial pattern of pellet-directed endpoint control, with additional later-emerging changes in reach timing. This pattern supports the interpretation that the LRN contributes importantly to the refinement and stabilization of skilled forelimb behavior, helping to ensure that the reaches are executed in a consistent manner. More broadly, these findings support the view that disruption of LRN-associated circuitry can degrade dexterous movement by impairing movement refinement and trial-by-trial control, even when the basic structure of the reach remains intact.

## Synthesis

Reviewing Editor: David Franklin, Technische Universitat Munchen Decisions are customarily a result of the Reviewing Editor and the peer reviewers coming together and discussing their recommendations until a consensus is reached. When revisions are invited, a fact-based synthesis statement explaining their decision and outlining what is needed to prepare a revision will be listed below. The following reviewer(s) agreed to reveal their identity: NONE.

Your manuscript has now been reviewed by two experts in the field. They both appreciate the scientific contributions of the manuscript, showing that the LRN is needed for fine end-point motor control and stabilization. However, we all see a few areas in which the current manuscript could be strengthened, and hope that you will be able to address these issues in a revision. The reviewers have some further suggestions on plots and analysis that could be added which might further strengthen the results. I have compiled the comments of both reviewers into a single decision letter below. Please revise the manuscript to address their suggestions and concerns.

Comments from the reviewers

This manuscript examines the effect of lesions the the lateral reticular nucleus (LRN) on precise, voluntary forelimb movements in rats. Animals were trained to reach and grasp a food pellet as experimenters measured forelimb kinematics with a high-speed video system. In the treatment group, VGLUT1 neurons in the LRN were ablated with diphtheria toxin. Following this lesion, gross features of the movement remained intact, but the spatial dispersion of the paw at the reach endpoint increased. The authors conclude that the LRN contributes to the refinement and stabilization of voluntary forelimb movements. The combination of the ablation surgeries and the fine-grained kinematic analysis provide an important insight into the functionality of the LRN, which, as the authors note, is well positioned to relay motor-related information. This is a solid manuscript which will be of interest to the motor systems community. It is well-written and clear, and the data support the conclusions.

Major Comments

One concern is the small sample size used, as I am not sure how strong of a statement can be made from this. However, I would not require the authors to conduct more experiments, as they also correctly state that their study provides a new model for investigating LRN-dependent control in future studies.

There are already some differences between the experimental and the control group before surgical intervention. I would like for the authors to discuss this.

The manuscript focuses on the wrist trajectory, but are the authors also able to resolve joint angles? If so, it might be interesting to see whether control animals are able to exploit redundancy to achieve endpoint precision (c.f. Todorov &Jordan, Nature Neuro 2002), and whether this ability is impaired in the lesion group. Along similar lines, the authors measured forelimb and hindlimb kinematics; does the variability in the initial posture at reach onset differ between groups?

It would be useful to show more single-trial and single-animal data, especially for the key dispersion result in Fig. 4. This figure is a bit confusing, as there are only 4-11 observations in the control condition and 6-33 in the treatment on each day; do these correspond to (1) individual reaches for an example animal, (2) pooled individual reaches for all animals, or (3) within-animal averages? The text and legend suggest (2), but as few as four reaches pooled across all rats might not be enough to accurately estimate the covariance. In general, the manuscript could be more transparent about animal and trial numbers throughout the text, figures, and figure legends.

Quantification of dispersion with covariance ellipses is not unreasonable, but I wonder whether additional measures and plots would be useful (e.g., samplewise standard deviation as a function of time within the reach, convex hull volume after peeling, bagplots, etc.).

Why did the lesion group speed up on later days (Fig. 6)? This is a bit counterintuitive, and the authors may wish to elaborate further on their interpretation in the Discussion. It would also be useful to see velocity time series across the reach.

Minor comments

- In Fig. 2, I would recommend using the same y-axis scaling across days and dimensions.

- Typo: "in tact"

The results section could profit from subheadings

-Please consistently indicate the figure and figure panel that supports each statement where applicable throughout the text

Introduction

-Markerless pose estimation has already been successfully used in reach &grasp behavior in several animals, including rodents. The introduction could profit from mentioning some relevant work in this regard.

Material and Methods

Page 4:

-Text: "after our own RNAscope investigation that demonstrated that the neurons of the LRN express vGlut1 and do not express GABA (suggesting their excitatory nature), and that there are very few if any other parts of the caudal medulla containing neurons which express vGlut1, none of which are close enough to the LRN to be of concern for our particular injection surgery."

- Comment: Please show results of the indicated RNAscope investigation in the supplement, or cite the relevant publication

-The authors indicate that reach and grasp is skilled, yet do not provide any detail on how the animals were trained. Please provide information on the training paradigm.

-The authors chose to study only female animals, could they please comment on this?

-Please define "IBB".

-Please provide recording details in this section: what cameras were used? How many cameras were used and at which angles were they positioned. What is their framerate? This information can be found in distributed sections of the manuscript, so it would profit from describing this clearly in the material and methods section.

-Text: "which aligned well with previous cohorts"

-Comment: Sorry I do not fully understand what this means.

Figure 1:

-Please delete the hindlimb markers as they are completely irrelevant to this study.

-Please label the x-axis in (C).

-Please reduce transparency of region labels in (D).

Page 6:

-Please define how many animals were used for the ablation surgery and how many for the control surgery.

-Text: "using a custom made application" - what was used to create this application?

Page 7:

-The first sentence is a more elaborate description of the analysis pipeline, please fuse this with the paragraph above it.

Page 9:

-Please indicate the molarity for PB used- does this refer to 0.1M PB? Please sepcify.

Figure 2

-Please give units for the y axes of all plots where applicable.

-Please increase the size of the panel labels.

-Please move the y-axes labels closer to the panels they belong to.

Figure 3

-Please plot the thinner lines slightly less transparent and the thicker line slightly less thick, discerning individual trials is difficult.

-Please provide units along the axes, where possible.

Page 13:

-Text: "On the pre-surgical session, endpoint spread was already somewhat greater in the experimental group, although both groups remained relatively constrained in space"

-Comment: "Why could that be the case? Could the authors please address this in the Discussion and maybe briefly explain how they chose, which animal received the ablation injection vs. the control injection?"

Figure 4:

-Please adjust the axes so all ellipses fit onto the plots.

Page 16:

-Please adjust the colors of the overlaid points. The overlaid data points are difficult to see.

-Please double check, if all significant results indicated in the text are highlighted as such in Figure 5.

Page 18:

-The authors indicate that the temporal effects only emerged during later recording stages. In the discussion, could they please comment on why they think that would be the case?

Page 22:

-Text: "This interpretation is strengthened by the fact that the strongest and most consist difference across endpoint analyses was within increased dispersion."

-Comment: "consist" should read "consistent".

## Author Response

Synthesis of Reviews:

Computational Neuroscience Model Code Accessibility Comments for Author (Required):

N/A Synthesis Statement for Author (Required):

Your manuscript has now been reviewed by two experts in the field. They both appreciate the scientific contributions of the manuscript, showing that the LRN is needed for fine end-point motor control and stabilization. However, we all see a few areas in which the current manuscript could be strengthened, and hope that you will be able to address these issues in a revision. The reviewers have some further suggestions on plots and analysis that could be added which might further strengthen the results. I have compiled the comments of both reviewers into a single decision letter below. Please revise the manuscript to address their suggestions and concerns.

Comments from the reviewers This manuscript examines the effect of lesions the the lateral reticular nucleus (LRN) on precise, voluntary forelimb movements in rats. Animals were trained to reach and grasp a food pellet as experimenters measured forelimb kinematics with a high-speed video system. In the treatment group, VGLUT1 neurons in the LRN were ablated with diphtheria toxin. Following this lesion, gross features of the movement remained intact, but the spatial dispersion of the paw at the reach endpoint increased. The authors conclude that the LRN contributes to the refinement and stabilization of voluntary forelimb movements. The combination of the ablation surgeries and the fine-grained kinematic analysis provide an important insight into the functionality of the LRN, which, as the authors note, is well positioned to relay motor-related information. This is a solid manuscript which will be of interest to the motor systems community. It is well-written and clear, and the data support the conclusions.

We thank the reviewers and the editors for taking the time to review our manuscript. We have worked to address the points raised by the reviewers, which were very insightful and we believe have strengthened the paper. Our additions and changes are highlighted in yellow in the revised manuscript. As such, we do not copy the text of each change into the response letter here, but refer the reviewer to the section where each change has been made.

Major Comments One concern is the small sample size used, as I am not sure how strong of a statement can be made from this. However, I would not require the authors to conduct more experiments, as they also correctly state that their study provides a new model for investigating LRN-dependent control in future studies.

There are already some differences between the experimental and the control group before surgical intervention. I would like for the authors to discuss this.

We concur that the sample size is small, but are glad that the reviewer appreciates the new model and we believe it is an important first step despite the small sample. We've inserted further explanations in Section 3.1 and another smaller explanation with Section 3.2 discussing the pre-lesional differences. In summary, these Results sections now explicitly note these pre-lesional differences, and here and in the subsequent discussion we take them into account by not drawing conclusions about a role for the LRN in producing them.

The manuscript focuses on the wrist trajectory, but are the authors also able to resolve joint angles? If so, it might be interesting to see whether control animals are able to exploit redundancy to achieve endpoint precision (c.f. Todorov &Jordan, Nature Neuro 2002), and whether this ability is impaired in the lesion group. Along similar lines, the authors measured forelimb and hindlimb kinematics; does the variability in the initial posture at reach onset differ between groups? We thank the reviewer for this thoughtful suggestion. We agree that determining whether animals exploit multi-joint redundancy to stabilize endpoint position would provide important insight into the contribution of the LRN to skilled-reaching control. The present study was not designed or sufficiently powered to perform a complete analysis of coordination across all forelimb, trunk, and postural degrees of freedom. Nevertheless, we used the available three-dimensional tracking data to perform an exploratory elbow-angle analysis.

Three-dimensional elbow angle was defined as the included angle between the shoulder-to-elbow and wrist-to-elbow vectors. Elbow-angle waveforms were calculated across the analyzed reach interval, normalized from 0\% to 100\% of reach time, and first averaged across reaches within each animal and recording day. The resulting animal-level mean waveforms were then compared between control and experimental groups using independent two-sample one-dimensional SPM\{t\} analyses performed separately at 12 days pre-surgery, 11 days post-surgery, 32 days post-surgery, and 39 days post-surgery.

No significant control--experimental differences or suprathreshold SPM clusters were detected at any of the four recording days. Elbow configuration at reach onset likewise did not show a detectable group difference. These results are now presented in Extended Data Figure 2-1 and described in Section 3.1, Bilateral LRN ablation preserves the gross structure of skilled reaching.

The absence of a detectable elbow-angle difference does not conflict with the observed endpoint differences. Endpoint position depends on the combined position and configuration of the shoulder, elbow, wrist, trunk, and body relative to the pellet. Similar elbow angles could therefore occur alongside differences in shoulder position, trunk posture, wrist configuration, or coordination among multiple joints. Within the resolution and statistical power of the present dataset, we therefore conclude that the endpoint deficits were not accompanied by a detectable group-level alteration in elbow-angle trajectory, while acknowledging that a more complete test of multi-joint redundancy will require larger cohorts and expanded tracking of proximal and postural landmarks.

We appreciate this suggestion because it allowed us to distinguish the observed endpoint-control deficit from a generalized alteration in elbow-angle trajectory.

It would be useful to show more single-trial and single-animal data, especially for the key dispersion result in Fig. 4. This figure is a bit confusing, as there are only 4-11 observations in the control condition and 6-33 in the treatment on each day; do these correspond to (1) individual reaches for an example animal, (2) pooled individual reaches for all animals, or (3) within-animal averages? The text and legend suggest (2), but as few as four reaches pooled across all rats might not be enough to accurately estimate the covariance. In general, the manuscript could be more transparent about animal and trial numbers throughout the text, figures, and figure legends.

Quantification of dispersion with covariance ellipses is not unreasonable, but I wonder whether additional measures and plots would be useful (e.g., samplewise standard deviation as a function of time within the reach, convex hull volume after peeling, bagplots, etc.).

We thank the reviewer for raising these points. The observations in the endpoint covariance figure are pooled individual reaches from all animals within each group and recording day. They are not reaches from a single example animal or within-animal averages. We agree that this was not clear enough in the original figure.

To improve transparency, we revised the endpoint covariance figure so that symbol shape and point shading identify the individual animal contributing each reach. We also revised the figure caption and Results text to report the number of animals and reaches represented in each group and recording day.

While preparing the additional endpoint visualizations suggested by the reviewer, we also identified a plotting-specific discrepancy in the number of reaches displayed in the original covariance figure. The reconstruction and broader analysis pipeline identified 157 eligible reaches, but the endpoint plotting table contained only 112 observations. This occurred because the plotting code required an exact match to the duration-derived endpoint frame and silently omitted a reach when the trial name did not match exactly, when the requested frame was unavailable, or when the MCP or pellet coordinate was briefly missing at that frame.

We corrected the plotting pipeline by standardizing trial-name matching, using the duration-derived endpoint frame when available, applying a trajectory-based endpoint fallback when needed, and selecting the nearest valid frame when the intended endpoint frame contained a brief tracking gap. The revised covariance and bagplot figures now include the complete eligible endpoint population.

This issue was limited to the endpoint table used for the dispersion visualizations. The inferential statistics and the other manuscript figures were generated through separate pipelines using the complete datasets and were therefore unaffected. Restoring the omitted reaches appropriately changed the displayed means, covariance boundaries, bag shapes, and sample counts, but it did not change the statistical results or the overall interpretation of the study. We also added a trial-level inclusion audit to the revised plotting pipeline to prevent silent omissions in future processing.

We appreciated the reviewer's suggestion to consider additional ways of visualizing endpoint dispersion. We therefore added bagplots as Extended Data Figure 4-1. Each symbol represents the endpoint of one reach, and symbol shape identifies the animal contributing that observation. The shaded bag contains the central approximately 50% of the distribution according to Tukey data depth, while the surrounding fence provides a robust view of the broader bivariate distribution. Points outside the fence remain visible and were not automatically treated as excluded observations. We present these bagplots as descriptive complements to the covariance ellipses rather than as a separate inferential test.

We also added a samplewise measure of trajectory variability as Extended Data Figure 3-1. For each animal and recording day, we calculated the three-dimensional radial standard deviation of the pellet-centered MCP trajectory across repeated reaches at each normalized reach timepoint. The individual curves represent the radial-SD waveform calculated separately for each contributing animal, while the group curves represent the mean of those animal-level waveforms. The shaded regions represent mean {plus minus} SEM across animals. These bands reflect uncertainty in the estimated group mean arising from variation among animals. They do not represent excluded observations, percentile envelopes, or the full trial-to-trial variability within each group. Because SEM depends on both between-animal variation and the number of contributing animals, we interpret these bands cautiously given the small cohort.

Independent two-sample SPM{t} analyses were performed on the animal-level radial-SD waveforms separately at each recording day using α = 0.05. No significant control-experimental differences or suprathreshold clusters were detected at any recording day.

We also considered the reviewer's suggestion to calculate convex-hull volume after peeling. Given the relatively small number of observations in some day-by-group subsets, however, the resulting hulls would be highly sensitive to individual points and would not provide a stable or comparable summary across all recording days. We therefore prioritized bagplots, which provide a more robust description of the central distribution while still displaying the full set of peripheral observations.

We appreciate the reviewer encouraging us to examine the dispersion results in greater detail. These additions improved the transparency of the animal and trial structure and provided complementary views of endpoint and trajectory variability.

Why did the lesion group speed up on later days (Fig. 6)? This is a bit counterintuitive, and the authors may wish to elaborate further on their interpretation in the Discussion. It would also be useful to see velocity time series across the reach.

We thank the reviewer for this suggestion. We agreed that showing velocity across the reach would help place the later reduction in reach duration in better context.

We therefore added three-dimensional velocity waveforms across normalized reach time as Extended Figure 6-1. Velocity was calculated from the reconstructed forelimb trajectories and expressed in millimeters per second from 0% to 100% of the analyzed reach interval. Trial-level waveforms were first averaged within animal and then summarized by group. Thin gray curves show the individual animal-level mean waveforms, thick blue and orange curves show the control and experimental group means, and the shaded bands represent mean {plus minus} SEM across animals. These bands reflect uncertainty in the estimated group mean arising from between-animal variation rather than trial-level variability.

We performed independent two-sample SPM{t} analyses on the animal-level mean velocity waveforms separately at each recording day using α = 0.05. No significant control-experimental differences or suprathreshold clusters were detected at 12 days pre-surgery, 11 days post-surgery, 32 days post-surgery, or 39 days post-surgery. The rat-level comparisons included two control animals and three experimental animals at each recording day.

We also ran an exploratory trial-level SPM analysis using all contributing reaches. This analysis reached the same conclusion, with no significant clusters at any recording day. The trial counts were control n = 13 and experimental n = 44 at 12 days pre-surgery; control n = 12 and experimental n = 26 at 11 days post-surgery; control n = 14 and experimental n = 12 at 32 days post-surgery; and control n = 8 and experimental n = 28 at 39 days post-surgery. Because repeated reaches from the same animal are not fully independent, we treat the animal-level analysis as the primary inferential result and the trial-level analysis as exploratory.

Although the visible group separations were not statistically significant, the waveforms still provide useful descriptive context by showing where within the reach the group mean profiles begin to diverge. We have therefore been careful not to present the velocity waveforms as independent evidence of a significant group effect.

We also expanded the Discussion to clarify that the later reduction in reach duration may reflect altered temporal regulation rather than improved motor performance. One possible interpretation is that the LRN contributed to regulating or slowing portions of the reach while sensorimotor information is being integrated to stabilize endpoint placement. Reduced LRN function could therefore allow a faster movement that is also less consistently controlled. We present this interpretation cautiously, but it is consistent with the combination of shorter reach duration and greater endpoint dispersion.

We also clarified why the timing effect may become more evident at later recording stages. The DTA-based ablation develops over the course of viral expression and subsequent neuronal degeneration rather than producing an immediate focal lesion. Later behavioral effects may therefore reflect the progressive development of the ablation and its downstream circuit consequences.

We appreciate the reviewer encouraging us to examine the full velocity profile, as it helped us present the timing result with more context and appropriate caution.

Minor comments - In Fig. 2, I would recommend using the same y-axis scaling across days and dimensions.

Completed. Within each coordinate dimension, the y-axis scaling was standardized across recording days to facilitate direct comparison, and units are now shown on all applicable axes.

- Typo: "in tact" Corrected. Highlighted on page 22.

-The results section could profit from subheadings The Results section was reorganized using the following subheadings:

Section 3.1, "Bilateral LRN ablation preserves the gross structure of skilled reaching" Section 3.2, "LRN ablation increases endpoint dispersion without eliminating pellet-directed reaching" Section 3.3, "Quantitative feature analysis identifies endpoint spread as the clearest lesion-related deficit" Section 3.4, "Reach-duration differences emerge later than endpoint precision deficits" -Please consistently indicate the figure and figure panel that supports each statement where applicable throughout the text Revised. We added figure and panel references throughout the Results and Discussion wherever specific claims are made. Trajectory preservation is now tied to Figure 2A-L and Figure 3A-D; endpoint covariance to Figure 4A-H; quantitative endpoint and variability features to Figure 5A-E; and reach-duration effects to Figure 6.

Introduction -Markerless pose estimation has already been successfully used in reach &grasp behavior in several animals, including rodents. The introduction could profit from mentioning some relevant work in this regard.

Added. We thank the reviewer for noting this.

Material and Methods Page 4:

-Text: "after our own RNAscope investigation that demonstrated that the neurons of the LRN express vGlut1 and do not express GABA (suggesting their excitatory nature), and that there are very few if any other parts of the caudal medulla containing neurons which express vGlut1, none of which are close enough to the LRN to be of concern for our particular injection surgery." - Comment: Please show results of the indicated RNAscope investigation in the supplement, or cite the relevant publication Added. Page 4, Section 2 Materials and Methods, Subsection 2.1 Subjects. The rationale for using the vGlut1-Cre line and the RNAscope expression findings are now described in Section 2.1, Subjects. The corresponding vGlut1 and GABA images are presented in Extended Data Figures 1-1 and 1-2.

-The authors indicate that reach and grasp is skilled, yet do not provide any detail on how the animals were trained. Please provide information on the training paradigm.

Page 4, Section 2.2. Added description of the training paradigm used for the rodents.

-The authors chose to study only female animals, could they please comment on this? Explanation added in Section 2.1. Only female animals proceeded to surgery and final analysis to maintain consistency within the available vGlut1-Cre breeding cohort and to avoid introducing sex as an additional experimental variable in this initial study. We now state explicitly that the findings should therefore be interpreted as specific to adult female Long-Evans rats.

-Please define "IBB".

Page 4, section 2.2. Defined IBB and now include relevant citation. The reaching-training paradigm and the definition of the Irvine, Beatties, and Bresnahan forelimb scale are now provided in Section 2.2, Pre-operative Behavioral Assessment and Reaching Training.

-Please provide recording details in this section: what cameras were used? How many cameras were used and at which angles were they positioned. What is their framerate? This information can be found in distributed sections of the manuscript, so it would profit from describing this clearly in the material and methods section.

We thank the reviewer for this suggestion. We added a paragraph explaining this. Camera number, arrangement, recording rate, image resolution, calibration, and DeepLabCut processing details are now provided in Section 2.4, Automatic 2D Marker Tracking Using DeepLabCut.

-Text: "which aligned well with previous cohorts" -Comment: Sorry I do not fully understand what this means.

Revised. We removed the unclear phrase "which aligned well with previous cohorts." Animal enrollment, behavioral screening, viral assignment, surgical-group numbers, and final cohort composition are now described in Sections 2.1-2.3. Random assignment to the FLEX-DTA or AAV2-hSyn-mCherry control-virus group is stated explicitly in Section 2.3, Lateral Reticular Nucleus Injection Surgery.

Figure 1:

-Please delete the hindlimb markers as they are completely irrelevant to this study.

Completed. Hindlimb markers were removed from Figure 1B, and the figure caption was revised to describe only the forelimb and pellet markers used in this study.

-Please label the x-axis in (C).

Completed. The x-axis in Figure 1C is now labeled "Month." -Please reduce transparency of region labels in (D).

Done. Increased opacity from 40% to 80%.

-Please define how many animals were used for the ablation surgery and how many for the control surgery.

Fourteen animals, including nine females and five males, entered skilled-reaching training. Six female animals met the behavioral training criterion and were selected for surgery. These six animals were randomly assigned to receive either the experimental FLEX-DTA construct or the AAV2-hSyn-mCherry control construct. Four animals received AAV2-mCherry-FLEX-DTA and two received AAV2-hSyn-mCherry. One FLEX-DTA-treated animal was subsequently excluded because of irregular reaching behavior, leaving five animals in the final kinematic cohort: three experimental animals and two control animals. These numbers and the random-assignment procedure are now described in Sections 2.1-2.3.

-Text: "using a custom made application" - what was used to create this application? Revised. We clarified that the software environment used to create the custom marker-review application was a Python (citation?) script that uses the QT framework (citation?) to make an interactive graphical user interface (GUI).

Page 7:

-The first sentence is a more elaborate description of the analysis pipeline, please fuse this with the paragraph above it.

Added. The end of Section 2.5 has been adjusted to fully explain the pipeline, and Section 2.6 has been revised to focus less on the pipeline and more on statistical analyses.

Page 9:

-Please indicate the molarity for PB used- does this refer to 0.1M PB? Please sepcify.

The use of 0.1 M phosphate buffer is now specified in Section 2.7, Perfusion and Dissection.

Figure 2 -Please give units for the y axes of all plots where applicable.

-Please increase the size of the panel labels.

-Please move the y-axes labels closer to the panels they belong to.

Fixed. All panel label text size was increased. Y-axis labels were increased in size &located closer to the graphics they correspond to. Units (mm) are now included.

Figure 3 -Please plot the thinner lines slightly less transparent and the thicker line slightly less thick, discerning individual trials is difficult.

-Please provide units along the axes, where possible.

Fixed. All panel label text size was increased and units (mm) are included.

Page 13:

-Text: "On the pre-surgical session, endpoint spread was already somewhat greater in the experimental group, although both groups remained relatively constrained in space" -Comment: "Why could that be the case? Could the authors please address this in the Discussion and maybe briefly explain how they chose, which animal received the ablation injection vs. the control injection?" Revised. The presurgical trajectory and endpoint differences are acknowledged in Sections 3.1 and 3.2 and are discussed explicitly as a limitation in Section 4, Discussion. The revised text clarifies that animals were not assigned according to presurgical endpoint position or dispersion and that these baseline differences are not attributed to LRN loss.

Figure 4:

-Please adjust the axes so all ellipses fit onto the plots.

Adjusted. Ellipses have been zoomed out so that all are fully visible in each panel.

Page 16:

-Please adjust the colors of the overlaid points. The overlaid data points are difficult to see.

-Please double check, if all significant results indicated in the text are highlighted as such in Figure 5.

Corrected. The colors have been outlined in a neutral color to allow them to stand out against the orange/blue backgrounds.

Page 18:

-The authors indicate that the temporal effects only emerged during later recording stages. In the discussion, could they please comment on why they think that would be the case? Comment added. We now cite related work documenting that neurodegenerative targeted lesions with DTA take approximately 3 weeks for lesions to fully appear.

Page 22:

-Text: "This interpretation is strengthened by the fact that the strongest and most consist difference across endpoint analyses was within increased dispersion." -Comment: "consist" should read "consistent".

Corrected. "Consist" was changed to "Consistent", and the sentence was revised for clarity.
